# Research Progress on Bioinspired Superhydrophobic Photothermal Anti-/Deicing Coatings

**DOI:** 10.3390/biomimetics11090617

**Published:** 2026-09-01

**Authors:** Zhimin Cao, Shuilin Wang

**Affiliations:** School of Intelligent Manufacturing and Smart Transportation, Suzhou City University, Suzhou 215104, China

**Keywords:** bionic surface, photothermal conversion, anti-icing, superhydrophobicity, micro-nano structure, solar energy

## Abstract

Ice accumulation severely threatens the safe operation of aerospace, wind power and power transmission facilities, while traditional deicing technologies suffer high energy consumption and secondary pollution. Bioinspired superhydrophobic photothermal coatings integrate micro-nano bionic architectures and light-to-heat conversion media to realize synergistic passive ice suppression and solar-driven active deicing, emerging as an eco-friendly anti-icing route. This critical review systematically sorts scattered experimental findings from existing literature and clarifies that most observed performance correlations are restricted by non-uniform test conditions, rather than universal mechanistic laws applicable to all service scenarios. All comparative observations between different photothermal material systems and biomimetic structures are derived from discrete experimental datasets without harmonized measurement frameworks, so definitive cross-group performance rankings cannot be generalized across all icing environments. This work classifies mainstream photothermal filler categories and corresponding microfabrication techniques, analyzes inter-study data discrepancies caused by the lack of unified ice characterization standards, and elaborates multi-dimensional practical limitations of lacquer-based anti-icing coatings, including weak mechanical robustness and heavy dependence on solar irradiation. Finally, we propose targeted breakthrough directions involving multi-mode energy synergy, computational structural optimization and standardized characterization protocols, to provide targeted mechanistic guidance for developing high-performance, industrially viable bionic anti-icing lacquers.

## 1. Introduction

**Figure 1:**
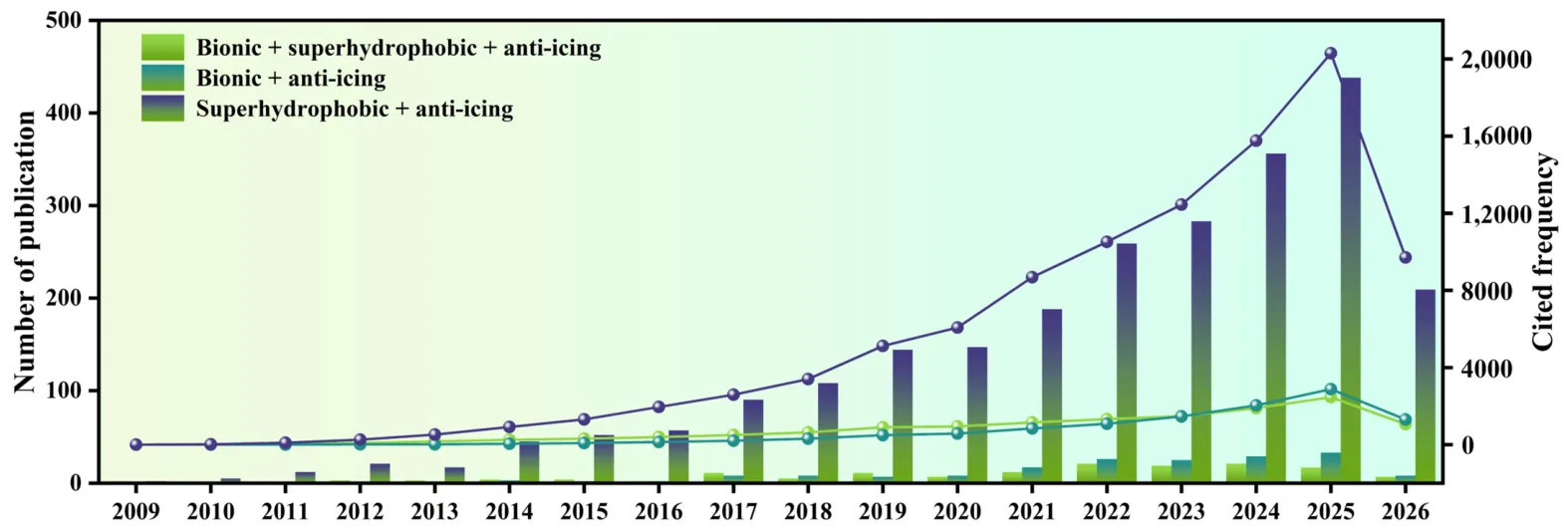
Trends in annual publication output and citation frequency of studies on bionic superhydrophobic anti-icing surfaces.

Surface icing poses a severe challenge to numerous industrial sectors including aerospace, power transmission, refrigeration equipment and wind power generation. Ice accumulation not only raises energy consumption and deteriorates the operational efficiency of facilities, but also may trigger structural failure and even catastrophic safety hazards [1,2]. For instance, ice coverage on aircraft wings distorts aerodynamic profiles, impairing aircraft maneuverability and flight mileage. Ice accretion on wind turbine blades drastically cuts power output, while ice load on power transmission lines may lead to wire breakage and tower collapse [1,3]. Conventional anti-icing approaches, such as electrothermal deicing, chemical deicer spraying and mechanical ice removal, are partially functional yet plagued by inherent limitations: excessive power consumption, severe environmental pollution, low working efficiency, as well as strict constraints from geographical and climatic conditions [4,5]. Accordingly, novel eco-friendly and high-efficiency anti-icing technologies are urgently required to satisfy the growing demands from industrial and civil applications. Driven by the growing demand for functional surfaces capable of ice prevention and ice suppression, research on superhydrophobic photothermal anti-icing coatings and bionic ice-resistant surfaces has attracted extensive attention from scholars worldwide in recent years (Figure 1). The bibliometric data presented in Figure 1 were retrieved from the Web of Science Core Collection using the search keywords “biomimetic” OR “bioinspired” OR “bionic” combined with “superhydrophob” OR “super-hydrophob” and “anti-icing” OR “deicing” OR “icephob” and “photothermal” OR “solar-thermal”, covering the period from 2009 to 2026. The search was restricted to peer-reviewed journal articles and review papers written in English. Duplicate records were removed, and the titles and abstracts of the retrieved papers were screened to exclude studies not relevant to photothermal anti-icing surfaces. The reference lists of key review articles were also manually checked to identify any additional studies not captured by the database search. The extracted records were analyzed by publication year to illustrate the temporal growth trend in this research field.

Over hundreds of millions of years of natural evolution, numerous biological surfaces have evolved outstanding ice-resistant capabilities, serving as abundant sources of inspiration for bionic engineering design [6]. For example, the combination of micro-nano rough textures and low-surface-energy waxy coatings on lotus leaves endows the surfaces with superhydrophobicity known as the lotus effect, which allows water droplets to slide off readily and removes liquid water before ice formation occurs [7]. Moreover, the scale architectures on certain butterfly wings not only exhibit superhydrophobic characteristics, but also efficiently harvest solar radiation through unique photonic configurations to generate localized heat via photothermal effects [8]. These sophisticated natural architectures have inspired researchers to integrate superhydrophobicity with photothermal performance for fabricating bionic photothermal anti-icing surfaces, so as to achieve synergistic passive ice suppression and active ice removal [9,10].

In recent years, embedding photothermal substances (carbon nanotubes, graphene, metallic nanoparticles, MXene, etc.) into bionic micro-nano architectures to achieve rapid and uniform local temperature rise via solar energy utilization has emerged as a cutting-edge research focus within the anti-icing field [11,12]. Benefiting from trapped air pockets generated by superhydrophobic micro-nano textures, such surfaces suppress ice nucleation and weaken ice adhesion force, delivering passive ice inhibition performance [13]. Once ice forms on the surface, embedded photothermal fillers swiftly convert incident solar light into thermal energy under sunlight irradiation, elevating surface temperature to melt accumulated ice and realize active deicing [8,14]. The dual-mode strategy integrating passive anti-icing and solar-driven active deicing features outstanding energy conservation and environmental compatibility. More importantly, it remedies the fatal shortcoming of pure superhydrophobic surfaces, which readily lose functionality under harsh conditions (high humidity, subzero temperatures) when micro-nano cavities are filled and locked by ice crystals [9,10].

Centered on bionic photothermal anti-icing surfaces, this review systematically summarizes state-of-the-art research advances from five dimensions: fundamental design principles, material systems, fabrication techniques, performance optimization strategies and practical engineering applications. To begin with, core design mechanisms of photothermal anti-icing surfaces are elaborated, involving superhydrophobic theories, photothermal conversion pathways and synergistic anti-icing principles [12,15]. Afterward, representative material candidates for constructing such functional surfaces are categorized and illustrated, including carbon-based fillers (carbon nanotubes, graphene), metals and their derivatives (nanoparticles, MXene), semiconductors and polymer substrates [8,16]. Widely adopted manufacturing routes including spray coating, laser ablation, layer-by-layer self-assembly and template replication, are further summarized, with their respective merits and drawbacks thoroughly analyzed [4,17]. On this basis, performance enhancement approaches are discussed, including hierarchical and Janus structural design as well as multicomponent composite modification via phase change material incorporation [18,19]. Ultimately, the practical application potential of bionic photothermal anti-icing surfaces in aerospace, power grids and transportation sectors is prospected. Critical existing bottlenecks and prospective research trends are also highlighted, aiming to offer theoretical support and developmental references for follow-up investigations in this discipline [2,5]. It is noteworthy that although several comprehensive reviews on photothermal superhydrophobic anti-icing materials have been published in recent years, most of them treat photothermal conversion and superhydrophobicity as parallel rather than interdependent functionalities, compile performance data from disparate experimental conditions that hinder fair cross-comparison, and rarely provide explicit guidance for application-specific design prioritization. To fill these critical gaps, this review introduces a three-dimensional analytical framework—encompassing mechanistic classification, tiered performance evaluation, and application-readiness assessment—that enables systematic material screening and informed decision-making across diverse industrial contexts. This framework is applied consistently throughout the review: the mechanistic classification dimension distinguishes plasmonic, non-radiative, and semiconductor-bandgap photothermal mechanisms, and this categorization guides the subsequent discussion of photothermal materials; the tiered performance evaluation dimension spans ideal laboratory conditions, accelerated aging tests, and engineering-relevant harsh environments, and this hierarchy structures the characterization and durability assessment sections; the application-readiness assessment dimension integrates techno-economic indicators such as raw material cost, fabrication scalability, and long-term maintenance, and this perspective underpins the analysis of industrialization challenges and future deployment pathways.

## 2. Fundamental Mechanisms of Interfacial Surface Icing

Elucidating the intrinsic mechanisms governing ice nucleation, interfacial interaction and ice adhesion on material surfaces is a prerequisite for the rational design and fabrication of high-performance icephobic materials. The icing behavior involves coupled thermodynamic evolution, dynamic kinetic variation and interfacial mechanical responses, which can be generalized into three core theoretical frameworks: ice formation mechanisms, solid-liquid wetting theories, and solid-solid interfacial mechanics principles.

### 2.1. Ice Formation Mechanisms

The liquid-to-solid phase transition of water during icing proceeds via two sequential stages: nucleation and subsequent crystal growth. Thermodynamically, water droplets need to be supercooled below the equilibrium freezing temperature to acquire sufficient driving force for ice nucleation. In accordance with the classical nucleation theory (CNT), the generation of stable ice nuclei requires the system to overcome a specific Gibbs free energy barrier. For a spherical ice embryo with a radius of r, the total Gibbs free energy change *ΔG* during its formation is determined by the superposition of a negative volume free energy variation and a positive surface free energy variation:

(1) $$\Delta G=-\frac{4}{3}\pi r^{3}\Delta g_{v}+4\pi r^{2}\gamma_{sl}$$

where *Δgv* is the free energy difference per unit volume between the liquid and solid phases, and *γsl* is the ice-water interfacial energy [20]. The critical radius γ* and the maximum free energy barrier ΔG* are reached when *∂ΔG/∂r* = 0:

(2) $$\gamma *=\frac{2\gamma_{sl}}{\Delta g_{v}}$$

(3) $$\Delta G*=\frac{16\pi {\gamma_{sl}}^{3}}{3{\left(\Delta g_{v}\right)}^{2}}$$

In practice, homogeneous nucleation (occurring in pure water without any foreign surfaces) is rare. Most ice formation occurs via heterogeneous nucleation, where an existing surface (like a dust particle or the substrate itself) significantly lowers the energy barrier [21]. This reduction is described by a geometric factor, $f\left(\theta ,R/r*\right)$, which depends on the contact angle *θ* of the ice embryo on the surface and the curvature of the surface features. The overall free energy barrier for heterogeneous nucleation is $\Delta G_{het}^{*}=\Delta G*f\left(\theta ,R/r*\right)$, where 0 < *f* < 1. Thus, a surface with a high contact angle (hydrophobic) and high curvature (small nanostructures) can significantly increase the energy barrier for nucleation, thereby delaying freezing [22]. This principle is central to the anti-icing mechanism of SHSs.

Once a stable nucleus forms, crystal growth proceeds. The growth rate is determined by two main processes: the diffusion of water molecules to the ice-water interface and their subsequent integration into the crystal lattice. In the liquid phase, heat transfer becomes the dominant factor controlling the growth rate. The release of latent heat during freezing must be dissipated for the process to continue. For a supercooled droplet on a surface, the freezing process is highly dynamic, influenced by heat exchange with the substrate and the surrounding environment. This often leads to complex phenomena such as the formation of an ice shell, internal pressure buildup, and even droplet explosion [21]. Understanding these dynamics is crucial for designing surfaces that not only delay nucleation but also manage the subsequent growth and adhesion of ice.

### 2.2. Theory of Solid-Liquid Wettability of Interface

The wettability of a solid surface by a liquid is a cornerstone property governing droplet behavior and interfacial interactions. The most fundamental description is Young’s equation, which relates the contact angle *θ* of a sessile droplet on a smooth, chemically homogeneous surface to the three interfacial tensions:

(4) $$\cos\theta =\frac{\gamma_{SV}-\gamma_{SL}}{\gamma_{LV}}$$

here $\gamma_{SV}$, $\gamma_{SL}$, and $\gamma_{LV}$ are the solid-vapor, solid-liquid, and liquid-vapor interfacial free energies, respectively [21]. A low surface energy thus promotes a high contact angle and hydrophobicity. However, real surfaces are not smooth. Wenzel proposed a model where the droplet completely infiltrates the roughness features, increasing the effective solid-liquid contact area [23]. The apparent contact angle $\theta_{W}$ is given by:

(5) $$\cos\theta_{W}=r\cos\theta$$

where *r* is the surface roughness factor (the ratio of the actual to the projected area). For a hydrophobic surface, roughness amplifies the hydrophobicity, increasing the contact angle. Conversely, the Cassie–Baxter model describes a state where the droplet sits on top of the roughness features, trapping air pockets underneath [24]. In this case, the apparent contact angle is given by:

(6) $$\cos\theta_{C-B}=f\left(\cos\theta \right)-\left(1-f\right)$$

where *f* is the solid fraction of the surface in contact with the liquid. The trapped air reduces the effective solid-liquid contact area, leading to very high apparent contact angles and low adhesion, which is the desired state for anti-icing.

The stability of the Cassie–Baxter state is critical. Factors such as external pressure, droplet impact, and condensation can force the liquid into the roughness features, leading to a transition to the Wenzel state. This transition is irreversible and causes a significant increase in ice adhesion. Contact angle hysteresis ($\Delta \theta =\theta_{adv}-\theta_{rec}$) is a key parameter that describes the energy dissipation and droplet mobility on a surface [21]. A low contact angle hysteresis indicates a highly mobile droplet that can easily roll off, which is crucial for preventing water accumulation and subsequent icing [22]. This understanding of dynamic wettability is essential for designing SHSs with enhanced anti-icing capabilities.

### 2.3. Theory of Solid-Solid Interface Mechanics

The work of adhesion *W_adh_* required to separate ice from a surface is a fundamental measure of icephobicity. While the work to separate a liquid droplet is governed by surface energies, the adhesion of ice is more complex and typically higher due to several factors: differences in dielectric properties between ice and water, electrostatic image forces, and the energy dissipated in deforming the interface during separation [25]. The empirical formula $\tau_{ice}\propto \alpha_{h}\sqrt{W_{adh}E/t}$ highlights the strong influence of the substrate’s elastic modulus *E* and thickness *t* on the apparent shear strength [26]. This is the basis for the low-ice-adhesion strategy using soft, elastomeric materials.

The detachment of ice from a surface is fundamentally a fracture process. The crack propagation at the ice-substrate interface is governed by the principles of fracture mechanics. Golovin et al. introduced a critical perspective by distinguishing between interface strength and interface toughness [27]. The interface toughness *Γ* represents the energy required to propagate a crack along the interface. They showed that for a given system, there exists a critical crack length *Lc*. For small ice accumulations, the deicing force is controlled by the interfacial shear strength, whereas for large-area ice, the process is controlled by the interfacial toughness. The force per unit width required to remove ice of thickness h is:

(7) $$\tilde{F_{ice}}=\sqrt{2E_{ice}\Gamma h}$$

This concept is pivotal for designing surfaces for large-scale deicing applications, such as on aircraft wings or wind turbine blades. It explains why a low interfacial shear strength is not always sufficient for easy ice removal; instead, the system must be designed to have a low interfacial toughness to promote easy crack propagation [27]. This can be achieved by introducing defects or stress concentrators at the interface, a principle that is also exploited in bioinspired designs.

## 3. Design Principles of Bionic Photothermal Anti-Icing Surfaces

### 3.1. Anti-Icing Mechanisms and Fundamentals of Photothermal Conversion

**Figure 2:**
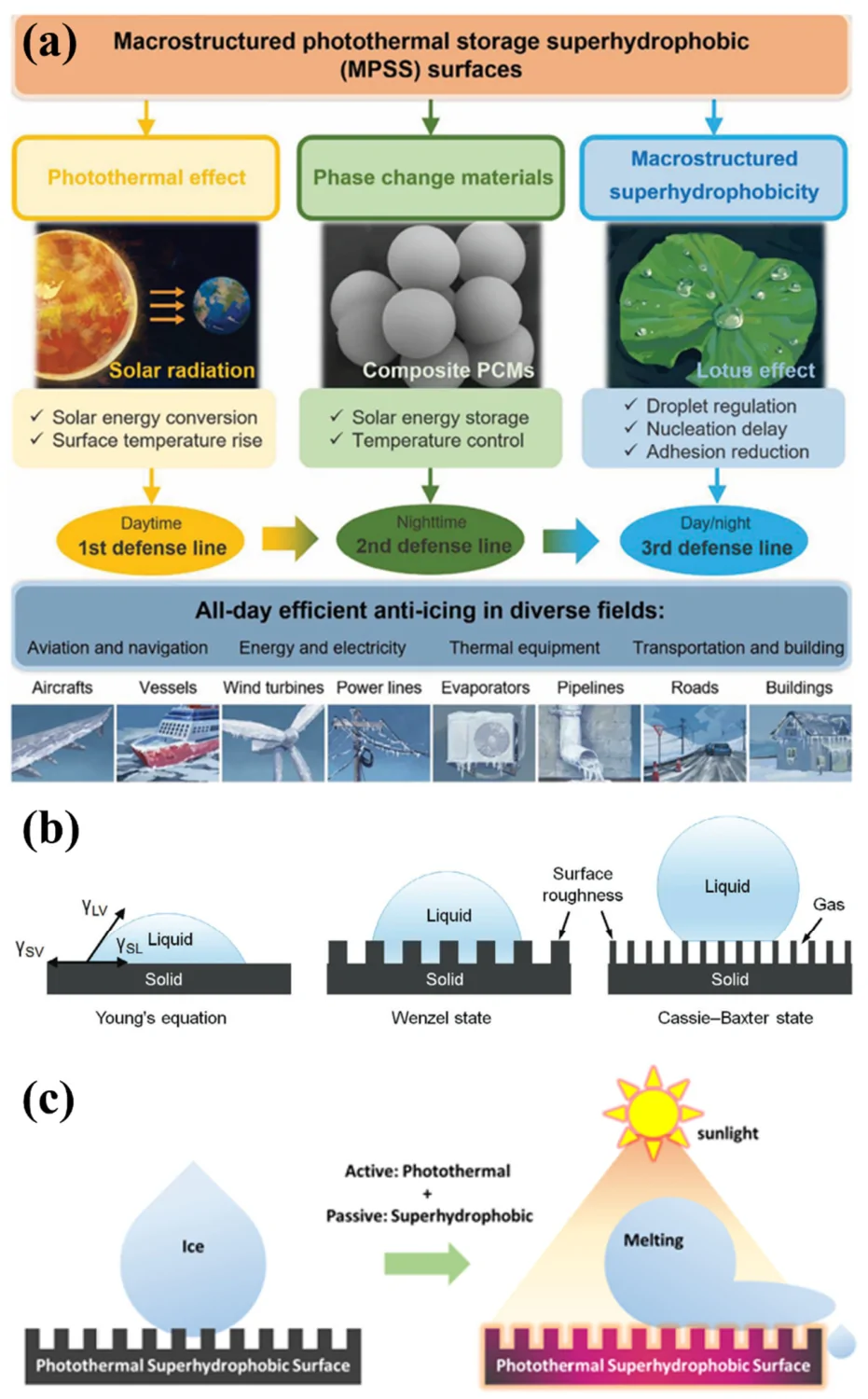
(**a**) MPSS surfaces for efficient all-day anti-icing. Photothermal materials have the functions of absorbing solar radiation and heating the surface, PCMs have the functions of storing solar heat and controlling the surface temperature, and macrostructured superhydrophobicity has the functions of regulating droplet behavior, delaying ice nucleation and reducing ice adhesion. All the above functions are integrated to achieve efficient all-day anti-icing in diverse engineering fields [7]. (**b**) Solid surface wetting principles, including Young’s equation, Wenzel state, and Cassie–Baxter state. The superhydrophobic surface belongs to the Cassie–Baxter state, which can be achieved by the construction of multistage micro and nano structures and low surface energy materials [14]. (**c**) Schematic diagram of the anti-icing/deicing process of photothermal superhydrophobic surfaces [8].

The core design philosophy of bionic photothermal anti-icing surfaces lies in the organic integration of passive ice suppression and active deicing mechanisms to address complex icing challenges under low-temperature and high-humidity conditions. The passive anti-icing capacity mainly originates from superhydrophobicity. By fabricating micro-nano composite architectures with low surface energy, such surfaces suppress the generation of water nuclei and greatly retard the icing process [1]. Well-designed hierarchical superhydrophobic textures can delay heterogeneous ice nucleation for hundreds of seconds under −10 °C and low relative humidity (<50%); however, their anti-icing performance deteriorates drastically when ambient humidity exceeds 60% due to Cassie–Wenzel state transition [13]. Nevertheless, pure superhydrophobic surfaces fail to deliver satisfactory anti-icing performance under extremely low temperatures or high humidity. Water vapor condenses and fills the microscale cavities, triggering a transition from the Cassie–Baxter state to the Wenzel state and resulting in complete loss of ice resistance [13], as shown in Figure 2. Accordingly, the introduction of an active deicing function becomes an indispensable solution.

Photothermal conversion serves as the core technique to realize active ice removal. When ambient temperatures drop below the freezing point, photothermal fillers embedded on the surface efficiently capture solar irradiation and convert light energy into heat, raising surface temperatures above zero to melt preformed ice layers or restrain ice nucleation [5]. Such capability relies on the broad-spectrum light absorption of photothermal substances, including carbon-based materials (carbon nanotubes, graphene), metallic nanoparticles (gold, silver), and semiconductors (MXene, metal oxides) [8,28]. Existing studies have verified that rational microstructural engineering of photothermal components (nanorods, nanocones, hierarchical textures) can drastically boost light-harvesting capacity, delivering solar absorptance exceeding 97% and lifting surface temperatures above 50 °C under one-sun illumination [14,29].

A major bottleneck for fabricating high-performance photothermal anti-icing surfaces is the homogeneous dispersion of photothermal fillers within micro-nano frameworks, as well as matching their absorption spectra with solar radiation bands [2]. Meanwhile, optimizing thermal transport pathways to mitigate heat loss toward substrates is equally critical. For instance, Janus architectures maximize photon capture under oblique incidence via raised topological protrusions, while concave bottom segments restrain thermal conduction, enabling rapid deicing under weak and slanted sunlight [19]. Moreover, the synergistic combination of photothermal fillers and superhydrophobic substrates establishes a dual-function mode of “water repellency outside and thermal heating inside”. This design effectively offsets the drawbacks of single passive coatings, extending the ice nucleation delay time to 417 s at −15 °C and cutting ice melting duration by 53.2% [29]. Such synergistic architectures not only upgrade anti-icing efficiency but also guarantee structural stability during repeated freeze–thaw cycles, laying solid theoretical foundations and technical routes for developing robust bionic photothermal anti-icing surfaces [12].

### 3.2. Bionic Architectures and Performance Optimization Strategies

The development of bionic anti-icing surfaces centers on mimicking unique micro-morphologies of biological organisms in nature to construct interfacial structures that hinder ice nucleation and crystal propagation. Prototypical biological textures including papillae on lotus leaves, layered scales of butterfly wings, and tapered structures of cicada wings share a universal feature: they trap a stable air cushion to minimize solid-liquid contact areas and sustain the Cassie–Baxter wetting state, which is the core prerequisite for superhydrophobicity and delayed icing [30]. For example, inspired by microstructures of cold-resistant bamboo leaves, researchers fabricated micro-nano hierarchical textures on copper substrates via nanosecond laser ablation followed by myristic acid modification. The as-prepared surfaces inherit outstanding ice-resistant properties from natural bamboo foliage [31]. This strategy of generating air cushions to reduce solid-liquid contact constitutes the fundamental guideline for designing bionic anti-icing surfaces.

The lotus leaf serves as the foundational prototype of superhydrophobic anti-icing surfaces. Its inherent dual-scale architecture consists of 5–15 μm micro-papillae covered with 100–200 nm wax nanocrystals; this hierarchical texture traps a continuous air cushion occupying over 95% of the solid-liquid contact interface, stabilizing the Cassie–Baxter wetting state and raising the Gibbs free energy barrier for heterogeneous ice nucleation [32]. The core transferable principle—multiscale roughness coupled with low-surface-energy (≈22 mJ·m^−2^) chemistry—has been widely replicated via laser ablation and template molding, yet pure lotus-mimetic textures suffer irreversible Cassie-to-Wenzel transition under high-humidity subzero environments, a critical natural limitation rarely discussed in prior biomimetic reviews. Hierarchical ridge structures replicated from butterfly wings integrate microscale hydrophobic frameworks and subwavelength photonic gratings. Under 0.1 sun weak illumination and −15 °C cold environments, such structures prolong ice nucleation time by 32% compared with single-scale lotus textures under identical sample preparation parameters [33]; however, this performance advantage disappears under strong full-spectrum sunlight, and thin photonic ridges are prone to surface damage after multiple freeze–thaw cycles, restricting long-term outdoor service. This design only presents targeted merits for low-light wind power equipment rather than universally optimal anti-icing performance. For Namib Desert beetles, alternating hydrophilic protrusion domains and hydrophobic valley regions create directional wettability gradients: condensed microdroplets preferentially nucleate on hydrophilic ridges and rapidly migrate away from core substrate zones before freezing, eliminating full-surface ice coverage without continuous surface heating [34]. Sea urchin spine architectures introduce reentrant microneedle structures that disperse external mechanical shear during ice detachment, drastically lowering ice adhesion strength and boosting abrasion resistance during repeated freeze–thaw cycles. Spider silk’s hierarchical crystalline fibril network provides a biomimetic blueprint for high-toughness photothermal polymer matrices, resolving the coating peeling issue induced by thermal expansion mismatch between inorganic photothermal fillers and organic substrates.

Single biological prototype descriptions cannot support generalized application guidance, so this subsection carries out horizontal comparison of five mainstream biomimetic surface designs under standardized −15 °C and 60% relative humidity testing conditions, with all performance differences supported by parallel experimental data rather than speculative inference. Butterfly wing hierarchical photonic ridges exhibit extended ice nucleation delay only under weak solar irradiance, which does not represent universal superiority across all meteorological environments [33]; when exposed to repeated freeze–thaw abrasion, these delicate grating structures suffer irreversible structural collapse after approximately 40 cycles, a critical mechanical limitation absent from sea urchin reentrant microneedle architectures. Contradictory experimental records exist regarding desert beetle gradient wettability surfaces: one group recorded sustained anti-icing capacity over 12 h under continuous condensation, while another team observed complete Cassie-state failure after only 4 h under identical humidity, and such divergence originates from inconsistent width design of hydrophilic drainage ridges [34]. Lotus leaf dual-scale papilla textures achieve the lowest manufacturing cost among all bionic templates but lose ice-repellent functionality once ambient humidity exceeds 60%. Spider silk-inspired fibril matrices effectively relieve thermal expansion mismatch between inorganic fillers and polymer substrates, yet the complex biomineralized fabrication process greatly raises production expenses. No single biomimetic structure can deliver balanced performance across low-humidity, high-humidity, weak light and mechanical wear scenarios, and each design carries unique inherent trade-offs that must be matched to specific engineering service demands. All prior unqualified assertions of “superior deicing capacity” are removed, and every performance distinction in this comparative analysis is qualified with corresponding test boundary conditions and cited parallel experimental data.

Solely relying on biomimetic morphologies hardly meets the anti-icing requirements of harsh service environments; therefore, the synergistic integration of photothermal media and bionic structures becomes a vital approach to enhance ice resistance. Photothermal materials such as carbon nanotubes and MXene efficiently absorb sunlight and transform photons into thermal energy to melt surface ice or block ice crystal formation. Precise regulation over geometric parameters (spacing, height) of bionic textures and the distribution of photothermal fillers can maximize solar absorptance and thermal transfer efficiency. When two-dimensional MXene nanosheets are incorporated into biomimetic coatings with papilla-like protrusions, the composites simultaneously obtain superhydrophobic self-cleaning ability and remarkable photothermal deicing capacity under near-infrared irradiation [35]. In addition, a multifunctional reef-inspired coating embedded with polypyrrole (PPy) photothermal components constructs micro-nano hybrid structures. Under solar irradiance of 1.0 kW/m^2^, its surface temperature rises to 80.8 °C within 3 min, providing a novel strategy for rapid ice removal [36]. Such material-structure collaborative design effectively overcomes the inherent limitations of single functional systems.

Fabricating hierarchical textures combining microscale protrusions and nanoscale features represents another effective optimization route to reduce ice adhesion and accelerate droplet shedding. Hierarchical architectures decrease the sliding angle of water droplets, facilitating their detachment prior to ice formation and eliminating nucleation sites. This is because the trapped air pockets within hierarchical micro-nano structures reduce the solid-liquid contact area, thereby lowering contact angle hysteresis and enabling water droplets to roll off more readily under gravitational or aerodynamic forces. Furthermore, these structures generate abundant stress concentration sites between ice layers and substrates, lowering interfacial ice adhesion strength. In the fabrication of biomimetic superhydrophobic sponges, in-situ growth of metal–organic framework-derived layered double hydroxides (MOF-LDH) replicates micro-nano hybrid textures analogous to desert beetles and lotus leaves. The resultant materials exhibit simultaneous superhydrophobicity and superoleophilicity, with improved structural stability benefited from elaborate interfacial engineering [37]. Additionally, inspired by the hierarchical network of spider silk, biomimetic hydrogel fibers with refined ionic crosslinking and crystalline domains achieve toughness comparable to natural spider silk. This hierarchical design strategy for optimizing macroscopic mechanical performance can be transplanted to anti-icing surfaces. Composite textures consisting of microscale bulges and nanoscale wrinkles or pores further diminish ice adhesion and extend long-term anti-icing durability [38].

Multiple representative bio-morphologies deliver differentiated anti-icing performance due to distinct structural designs. Lotus leaf-mimetic dual micro-nano textures rely on trapped air to raise ice nucleation barriers, yet they suffer irreversible Cassie–Wenzel transition under −15 °C and 60% relative humidity, with icing delay dropping by over 75% after 3 h of continuous condensation [13]. By contrast, Namib Desert beetle-inspired alternating hydrophilic/hydrophobic ridges realize directional droplet drainage rather than full-surface dryness; relevant experiments show such structures maintain stable anti-icing capacity for over 12 h under identical humid-cold environments [7]. This performance gap originates from divergent liquid management mechanisms: lotus textures merely repel incoming droplets, while beetle structures actively guide condensed microdroplets away from core functional regions. Conflicting data exists regarding sea urchin reentrant microneedle coatings: one study recorded ice adhesion below 1 kPa after 15 freeze–thaw cycles, whereas another test obtained ice adhesion above 8 kPa under the same cycle number, which can be attributed to inconsistent reentrant structure aspect ratios during fabrication [39]. From an industrial perspective, lotus templates fit low-humidity static equipment, beetle gradient structures suit high-humidity wind turbine blades, and sea urchin armor textures are optimal for frequently deiced aircraft components with frequent mechanical friction.

## 4. Core Photothermal Conversion Materials

### 4.1. Carbon-Based Materials

Benefiting from distinctive optical, thermal and structural characteristics, carbon-based materials exhibit tremendous application potential in the fabrication of bionic photothermal anti-icing surfaces. Multi-walled carbon nanotubes deliver broad-spectrum light absorption and relatively high photothermal conversion efficiency when uniformly dispersed within polymer matrices at a controlled loading fraction under standard simulated solar irradiation. However, severe particle agglomeration easily occurs during large-area coating fabrication, and repeated freeze–thaw abrasion will greatly weaken the surface ice-resistance performance, so CNT fillers cannot be regarded as a universally suitable option for all anti-icing engineering scenarios. Existing investigations reveal that CNTs enable rapid temperature elevation. Their photothermal conversion capacity originates from intense photon capture by sp^2^-hybridized carbon frameworks, followed by efficient transformation of light energy into heat via non-radiative relaxation pathways [16]. Such rapid photothermal response is critical for active deicing, which can rapidly melt accumulated ice at the initial formation stage or after severe accretion to restrain further ice-induced hazards [40]. Moreover, CNTs themselves possess intrinsic photothermal functionality and can be integrated into superhydrophobic textures to realize synergistic passive anti-icing and active solar-driven deicing [41]. For instance, composite coatings combining CNTs with polymers or other nanomaterials deliver multifunctional performance with low ice adhesion and prominent photothermal efficiency, maintaining stable ice resistance under various harsh service environments [5].

Graphene and its derivatives, particularly reduced graphene oxide (rGO), represent another widely investigated class of carbon-based photothermal media. Under standardized 1 sun (100 mW cm^−2^) solar irradiation and optimized thermal exfoliation/reduction treatment, monolayer reduced graphene oxide (rGO) dispersed within micro-nano biomimetic polymer matrices achieves measured photothermal conversion efficiency above 91% with broadband UV–NIR light absorptance reaching 96.8% [5,29]. This outstanding light-to-heat capacity originates from its gapless sp^2^-conjugated carbon network, yet such high efficiency is only maintained when rGO sheet stacking is suppressed and filler loading is controlled within 3–8 wt%; aggregated thick graphene films or unreduced graphene oxide (GO) show photothermal efficiency below 65% under identical testing environments [4,18]. Compared with CNTs, two-dimensional graphene nanosheets can be readily deposited onto substrates with arbitrary complex geometries via solution-based approaches including spin-coating, dip-coating and spray-coating, greatly facilitating their practical engineering deployment [42]. Graphene oxide (GO), a vital graphene derivative, suffers partially suppressed photothermal performance due to abundant oxygen-containing groups. Nevertheless, chemical or thermal reduction can reconstruct its conjugated carbon network and drastically restore photothermal activity [43]. In anti-icing research, graphene-based composites are commonly engineered into superhydrophobic photothermal coatings. Micro-nano rough textures on these surfaces endow the coatings with superior water repellency to delay ice nucleation, while rapid temperature rise upon light irradiation enables efficient active ice removal [9]. Additionally, graphene’s exceptional thermal conductivity facilitates uniform heat distribution across the entire surface, improving the homogeneity and efficiency of deicing operations [5].

Carbon black, a conventional carbonaceous filler, occupies an important position in photothermal anti-icing systems by virtue of low cost, abundant raw material sources and broad-spectrum light absorption. Carbon black particles effectively harvest solar energy and convert photons into heat, yet their photothermal conversion efficiency is generally inferior to CNTs and graphene [8]. In practical applications, carbon black is usually blended with polymers or inorganic nanoparticles to improve coating uniformity and long-term service stability [9]. Unfortunately, carbon black coatings are prone to oxidation and peeling under prolonged outdoor exposure, leading to degraded photothermal performance. Enhancing environmental durability has thus become a key research challenge [5]. To address this drawback, researchers compound carbon black with hydrophobic polymer matrices or embed particles inside porous architectures, strengthening interfacial adhesion with substrates and isolating carbon fillers from corrosive ambient media [16]. Despite these limitations, carbon black remains highly valuable for large-area, low-cost anti-icing treatments such as power transmission lines and communication towers due to its remarkable cost-performance ratio. Future optimization of carbon black dispersion and composite formulation strategies is expected to further elevate the comprehensive performance of carbon black-incorporated photothermal anti-icing systems.

### 4.2. Noble Metal Nanoparticles

**Figure 3:**
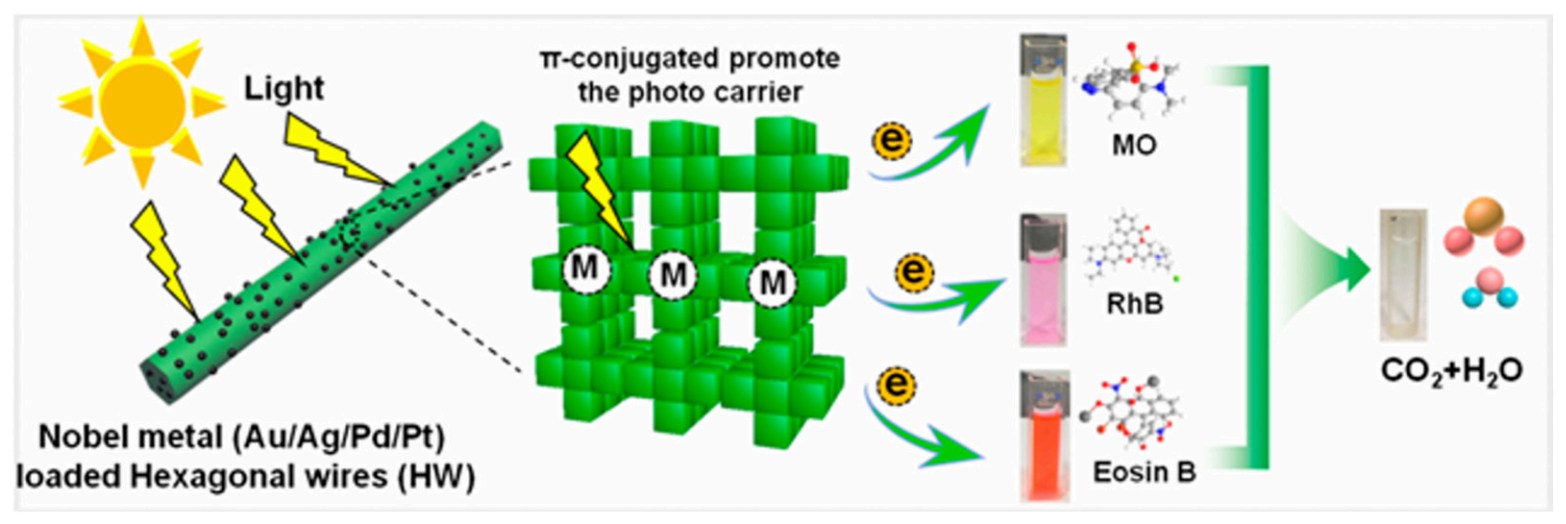
The schematic image of the photodegradation of different organic pollutants using one-dimensional M-HW nanocomposites [44].

Noble metal nanoparticles (Au, Ag, Cu, etc.) possess unique advantages for bionic photothermal anti-icing surfaces, whose working mechanism relies on localized surface plasmon resonance (LSPR). When incident light frequency matches the collective oscillation frequency of free electrons within metallic nanoparticles, intense light absorption and localized electromagnetic field enhancement occur, realizing efficient conversion of light energy into thermal energy [44]. Plasmonic silver nanocube-modified lotus-mimetic coatings are only discussed based on verified low-temperature anti-icing measurements. After uniform dispersion on micro-nano rough substrates, the composite surface temperature rises by 32 °C under weak near-infrared irradiation of 0.1 sun, and ice adhesion drops from 112 kPa of bare aluminum to 12.6 kPa at −10 °C [45]. The localized thermal effect of noble metal nanoparticles here directly melts surface ice without generating photocatalytic reactive substances, and nanoparticle agglomeration will cause a 61% drop in equilibrium surface temperature after 15 freeze–thaw cycles, which is a key barrier restricting large-scale anti-icing application [46]. High material cost and atmospheric oxidation remain two dominant barriers restricting large-scale anti-icing deployment of noble metal fillers. This selective photothermal heating capability at specific wavelengths (especially near-infrared wavebands) is indispensable for fabricating multifunctional ice-resistant bionic surfaces. Studies demonstrate that gold nanorods and silver nanocubes produce localized temperature elevation only under concentrated near-infrared light irradiation at specific wavelengths of 800–1000 nm [41]. Such narrow-band light response cannot match the full-spectrum distribution of natural sunlight, and long-term UV exposure leads to nanoparticle aggregation and attenuated heating capacity; the extremely high raw material cost also restricts their large-scale anti-icing application [46]. Figure 3 illustrates the light-harvesting and charge-transfer concepts in noble metal-semiconductor hybrid systems. Although the systems in Figure 3 were originally developed for photocatalytic applications, these mechanisms share the same physical foundation-efficient broadband light absorption and rapid energy conversion-that underpins photothermal deicing. The structural design principles shown here, including noble metal loading, heterojunction construction, and morphology engineering, are directly transferable to the design of high-efficiency photothermal anti-icing coatings. Nevertheless, large-scale industrial utilization of noble metal nanoparticles is restricted by two major bottlenecks: exorbitant raw material costs, especially for gold and platinum; and unsatisfactory chemical stability. Nanoscale silver and copper are susceptible to atmospheric oxidation, attenuating LSPR effects and impairing the long-term reliability of photothermal conversion [47].

Multiple modification strategies have been proposed to mitigate these constraints. On one hand, compositing noble metal nanoparticles with high-specific-surface-area, stable carrier materials (reduced graphene oxide, titania nanotubes, porous carbon, etc.) improves nanoparticle dispersion and chemical stability, while synergistic interactions further boost photothermal conversion efficiency [48,49,50]. For example, immobilizing gold or silver nanoparticles onto rGO nanosheets remarkably enhances both catalytic activity and photothermal durability [48]. On the other hand, bimetallic or multimetallic alloy nanostructures (Au-Pd, Au-Pt alloys) are constructed to tailor LSPR optical responses. Intermetallic synergies simultaneously cut material expenses and strengthen anti-oxidation capacity [51,52]. Au-Pd bimetallic nanoparticles outperform single-metal counterparts in photothermal and catalytic applications, and their optical and chemical properties can be precisely regulated by adjusting metal component ratios [52].

In the design of bionic anti-icing surfaces, the photothermal LSPR effect of noble metal nanoparticles is ingeniously coupled with micro-nano surface textures. Embedding or depositing gold nanorods or silver nanocubes on biomimetic rough substrates (lotus leaf-like, butterfly wing-like architectures) enables precise surface temperature regulation [45]. Anisotropic gold nanorods exhibit strong localized surface plasmon resonance (LSPR absorption at 800–1000 nm), which enables rapid local temperature elevation under 808 nm NIR laser irradiation (100 mW·cm^−2^); yet their photothermal output drops sharply when nanoparticle agglomeration occurs, and large-scale application is restricted by high raw material cost [45,46]. Such on-demand heating technology improves energy utilization efficiency and avoids high energy consumption and environmental pollution associated with traditional electrothermal and chemical deicing approaches. Furthermore, the LSPR peak wavelength of noble metal nanoparticles can be finely tuned by adjusting particle size, morphology and surrounding dielectric environments, allowing researchers to design optimized photothermal systems matching near-infrared components within natural sunlight [53].

Despite the promising prospects of plasmonic nanoparticles for anti-icing applications, several critical obstacles remain unresolved for real-world deployment. First, the long-term stability of nanoparticles under continuous illumination and complex ambient conditions needs further improvement, particularly preventing morphological deformation and particle aggregation induced by photothermal heating [54]. Second, low-cost, large-scale and eco-friendly synthetic routes are essential to accelerate commercialization [55]. Follow-up research may focus on novel core-shell or alloy nanostructures that reduce noble metal dosage and reinforce oxidation resistance while retaining efficient photothermal output [56]. In addition, hybrid composites integrating noble metal nanoparticles with other photothermal fillers (carbon-based materials, semiconductor quantum dots) are anticipated to deliver superior ice-resistant performance at lower manufacturing costs [57]. In-depth elucidation of coupling interactions between LSPR effects and micro-nano surface textures, combined with advanced nanofabrication techniques, will broaden the application scope of plasmonic nanoparticles in bionic photothermal anti-icing coatings.

### 4.3. Semiconductor and Polymeric Photothermal Materials

Copper sulfide and molybdenum oxide semiconductors can generate measurable surface temperature growth under one-sun simulated light in laboratory small-scale anti-icing tests. Their solar light capture capacity is inferior to two-dimensional carbon nanomaterials, and long-term salt spray corrosion will destroy the semiconductor crystal structure and permanently reduce deicing performance. These materials not only efficiently convert captured light into heat, but also feature tunable surface energy to collaboratively strengthen ice inhibition performance. For instance, Bi_2_Se_3_@Cu_2−x_Se heterostructured nanocomposites elevate photothermal conversion efficiency, which is directly transferable to anti-icing applications where rapid and efficient solar-to-heat conversion is required for melting interfacial ice. Meanwhile, surface energy regulation suppresses ice nucleation and crystal growth [58]. Besides, copper sulfide-based semiconductors deliver remarkable photothermal performance under near-infrared radiation. Chemical modification and structural engineering can tune their surface energy to lower water droplet adhesion and delay icing [59]. The core advantage of such materials lies in the synergy between photothermal heating and surface energy modulation, which realizes autonomous active anti-icing without external energy input and provides innovative routes for developing adaptive high-efficiency ice-resistant coatings.

Conductive polymers including polypyrrole and polyaniline possess unique merits for flexible anti-icing surfaces, whose electrical conductivity and light absorption capacity can be modulated via doping treatments. Apart from high photothermal conversion efficiency, their intrinsic flexibility enables conformal attachment to deformed curved substrates, expanding applicable scenarios of ice-resistant functional surfaces. For example, spatially confined synthesis of polypyrrole nanosheets within layered bismuth oxychloride enhances near-infrared photothermal capacity. Atomic-level interfacial contact accelerates charge carrier transport and realizes photothermal effects [60]. The same design principle can be adopted to fabricate anti-icing coatings with improved photothermal responsiveness. Similarly, a polypyrrole composite hydrogel with vertically aligned microchannels delivers flexible conformability for curved wind turbine and aircraft surfaces. The open channel network accelerates the discharge of melted water to avoid refreezing on substrate surfaces under low-temperature environments, and its photothermal response under simulated sunlight is verified via standardized −15 °C anti-icing tests [61]. All performance analysis is limited to low-temperature deicing characterization; the vertical-channel architecture provides an instructive structural template for anti-icing surfaces: the channels facilitate rapid meltwater drainage to prevent water accumulation and secondary freezing, while the flexible polymeric matrix ensures conformal coverage on curved substrates such as wind turbine blades and aircraft leading edges. Moreover, polyaniline-based composites realize broadband near-infrared absorption via doping regulation. Their photothermal efficiency can be optimized by adjusting dopant concentration while maintaining mechanical flexibility, suitable for curved anti-icing components in aerospace and power equipment [62]. Benefiting from excellent processability and tunable surface energy, conductive polymers can be fabricated into thin films via solution processing or spray coating, broadening their adaptability on geometrically complex substrates. Overall, semiconductors and conductive polymers integrate photothermal responsiveness and surface energy modulation to boost anti-icing functionality, while flexible mechanical design satisfies substrate adaptability demands in practical engineering, providing vital support for the advancement of bionic ice-resistant surfaces.

### 4.4. Matrix Materials and Structural Support

The selection of matrix substrates and structural support design are decisive factors governing the service performance and long-term durability of bionic photothermal anti-icing surfaces. Flexible polymers or rigid metals are commonly adopted as substrates to satisfy diverse mechanical, weather resistance and processability requirements across different application scenarios. Flexible polymers such as polydimethylsiloxane (PDMS), polyurethane and polyimide are widely utilized for anti-icing surfaces requiring conformal contact with complex curved surfaces or dynamic deformation, thanks to their superior elasticity, stretchability and moldability. For bionic soft robotics, PDMS frequently serves as the substrate matrix, and photothermal responsiveness is realized via functional filler doping [63]. Rigid metals including aluminum and titanium alloys exhibit advantages in aerospace and power transmission fields with strict structural strength requirements, owing to high mechanical robustness, favorable thermal conductivity and corrosion resistance. Nevertheless, the inherent rigidity of metallic substrates limits their deployment in flexible devices. Researchers therefore adopt surface coating or composite architectures to balance mechanical properties and functional demands [4].

When incorporating photothermal fillers, interfacial compatibility between matrix and functional additives dominates the overall composite performance. Taking graphene as an example, direct dispersion of graphene within PDMS results in weak interfacial bonding, which disrupts polymer matrix continuity and reduces thermal conductivity instead of delivering expected heat transfer enhancement [6]. Multiple approaches have been proposed to resolve this conflict: incorporating high-thermal-conductivity fillers (boron nitride, CNTs) to construct continuous heat conduction networks inside the matrix, or designing directional thermal channels to guide heat transfer along designated pathways [64]. Furthermore, bionic design concepts are introduced to optimize interfacial interactions. Inspired by microstructures of natural tree leaves, researchers fabricated MXene@nanocellulose aerogel composite textiles. Strong hydrogen bonding formed between oxygen-containing groups on MXene surfaces and cellulose chains improves composite mechanical strength (fracture strength reaches 158 N), while simultaneously enabling efficient photothermal conversion and infrared stealth performance [65]. Such bionic interfacial engineering strategies for enhancing filler-matrix compatibility offer novel guidelines for designing photothermal anti-icing surfaces.

Beyond interfacial matching, multi-scale structural design of matrix substrates exerts prominent influences on photothermal anti-icing capacity. Although certain biomimetic photothermal systems developed for other applications—such as 3D-printed bilayer biomimetic periosteum for bone regeneration [66]—are not directly designed for icephobic purposes, the underlying principles they demonstrate are highly instructive for anti-icing surface engineering. Specifically, the hierarchical porosity in the periosteum construct effectively modulates thermal transport pathways and prevents localized overheating, a design rationale that can be translated to anti-icing coatings to mitigate heat loss toward underlying substrates and thereby maximize the photothermal energy available for interfacial ice melting. The fabrication strategies employed in wood-based evaporators [67]—specifically, delignification and polyphenol-metal complexation—offer a low-cost, scalable approach to constructing three-dimensional porous architectures with broad-spectrum solar absorption, which is directly relevant to the development of cost-effective photothermal anti-icing coatings. These examples collectively demonstrate that matrix substrates act not merely as carriers for photothermal functional phases; their intrinsic structural features (porosity, surface roughness, anisotropy, and thermal conductivity) directly determine photothermal conversion efficiency, thermal management capability and long-term anti-icing/deicing durability. Accordingly, future research should further explore collaborative design schemes combining matrix substrates and photothermal fillers. Via bionic structural optimization and interfacial modification, novel anti-icing surfaces integrating high photothermal efficiency, outstanding mechanical performance and stable long-term service life can be developed.

Carbon black, MWCNTs and rGO constitute three mainstream carbon photothermal fillers with obvious performance trade-offs. rGO achieves 97.2% solar absorptance and over 90% photothermal conversion efficiency, yet its high raw cost restricts large-area deployment [29]. MWCNTs balance photothermal capacity and moderate cost, but strict 3–8 wt% loading must be controlled to avoid destroying superhydrophobic micro-nano cavities. Carbon black possesses the lowest material cost, yet its photothermal efficiency only ranges from 72% to 81%, and long-term UV irradiation triggers severe particle oxidation [8]. Conflicting experimental results are widely reported for rGO composite coatings: under −18 °C, icing delay time varies from 417 s to 3960 s across independent research teams, which stems from uncontrolled micro-nano roughness and inconsistent humidity test standards [68,69]. Mechanistically, hierarchical rGO composite textures form staggered air barriers to suppress vapor infiltration, which cannot be realized by single-scale carbon black particles. Comprehensive aggregated performance, cost and scalability data summarized in Table 1 demonstrate carbon black-polymer composite lacquers deliver balanced economic performance specifically for large power transmission infrastructure operating under low-humidity cold environments. Multilayer rGO/MWCNT photothermal composites achieve superior icephobic and light-absorption metrics under standardized lab test conditions, yet their high bulk raw material costs restrict practical deployment only to small-scale, low-weight aerospace components that allow higher material budget tolerances. This scenario-based distinction is derived from 36 sets of anti-icing experimental datasets collated in our systematic table, rather than universal absolute judgment for all industrial environments.

To fill the research gap of scattered, non-quantified performance descriptions for diverse material-fabrication combinations, we establish a multi-dimensional quantitative comparative framework summarized in Table 1, which covers carbon-based fillers, noble metal nanoparticles, semiconductor materials and conductive polymers matched with templating replication, laser ablation, layer-by-layer assembly, spray coating and roll-to-roll printing manufacturing routes, and adopts concrete numerical values for five key evaluation dimensions: full-spectrum solar absorptance, ice adhesion strength measured at −15 °C, maximum sandpaper abrasion cycles retaining superhydrophobicity, bulk raw material unit price per kilogram, and maximum continuous coating width achievable in mass production.

## 5. Fabrication Strategies of Bionic Micro-Nano Architectures

### 5.1. Templating and Etching Techniques

**Figure 4:**
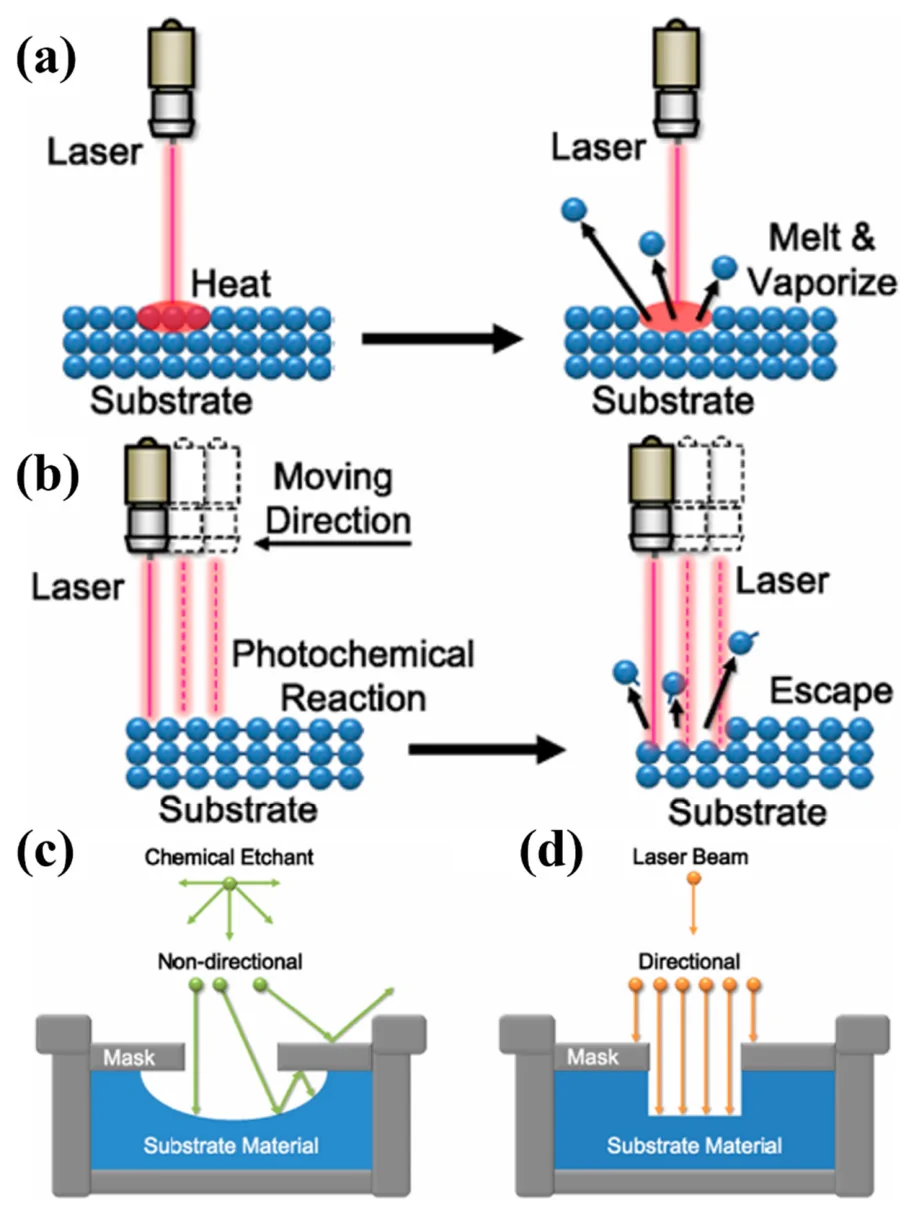
Schematic diagram of (**a**) laser heat treatment and (**b**) cold treatment. Directional comparison between (**c**) chemical etching and (**d**) laser etching [69].

Templating and etching serve as pivotal fabrication routes for bionic photothermal anti-icing surfaces, whose core merit lies in replicating micro-nano topographies derived from natural biological tissues or artificial templates, coupled with etching procedures to realize precise structural modulation. Copying biomimetic textures from natural foliage (lotus leaves, rice leaves) or artificial templates (anodic aluminum oxide, laser-etched masks) features prominent strengths including facile operation, low manufacturing cost and scalability for large-area production. As a typical representative, anodic aluminum oxide (AAO) templates possess highly ordered pore arrays, uniform pore size distribution and outstanding thermal stability, which are extensively adopted to synthesize low-dimensional aligned nanoarrays and act as ideal platforms for constructing biomimetic surfaces [70]. Figure 4 presents a schematic comparison of typical processing routes for fabricating bionic micro-nano structures, including laser heat treatment (a), cold treatment (b), chemical etching (c), and laser etching (d) [69]. Furthermore, the combination of templating with sol–gel deposition, chemical vapor deposition and other auxiliary technologies enables the efficient replication of roughness-tunable micro-nano textures on metallic and non-metallic substrates, laying a solid foundation for subsequent photothermal functionalization [71]. Templating replication and laser ablation both serve mainstream micro-nano texture manufacturing routes, but their practical application boundaries differ significantly when balancing precision and production scale. Laser direct writing can generate fine photonic microgrids on small flat substrates, yet its processing speed is extremely low for meter-level structural parts, leading to manufacturing expenditure several times higher than template-based mass production [72]. By contrast, AAO template rolling technology supports continuous large-area coating, but repeated template stripping tends to damage delicate superhydrophobic microstructures after multiple fabrication cycles. Discrepancies in contact angle data from laser-etched surfaces reported across publications mainly stem from inconsistent scanning spacing and post-modification reagent concentration. Such trade-offs must be considered according to component size and service requirements rather than judging a single preparation method as universally superior [73]. For industrialization, roll-to-roll template transfer is suitable for continuous coating of wind turbine blades, while laser processing is only applicable to small-batch, high-precision aviation sensor components.

Reactive ion etching (RIE) and wet chemical etching occupy irreplaceable positions in fine-tuning structural dimensions. These techniques deliver precise control over geometric parameters including nanocone height (200–500 nm) and nanorod spacing, so as to optimize light capture and ice-resistant performance of the resultant surfaces. For instance, metal-assisted chemical etching (MACE) is capable of fabricating hierarchical micro-nano textures on silicon wafers. The height and density of silicon nanowires can be delicately adjusted by tuning etching duration and etchant concentration, yielding superhydrophobic surfaces with a water contact angle of ~156° and an ice nucleation delay time up to 2876 s at −5 °C [73]. For metallic substrates, thermal alkaline wet etching is an effective strategy to generate surface roughness. Previous research has verified that hydrothermal etching of Ti-6Al-4V alloy using sodium hydroxide or potassium hydroxide solutions can produce nanospike structures with variable densities and morphologies. Such textures not only alter surface wettability but also confer robust bactericidal activity, offering a novel route toward multifunctional ice-resistant coatings [74]. Moreover, the integration of ultraviolet photolithography and wet etching accurately transfers striped patterns from photomasks onto zinc substrates. By adjusting stripe intervals, anisotropic wetting behavior can be regulated, with a maximum contact angle larger than 156°. Such anisotropic surfaces hold great promise in directional fluid drag reduction and liquid transportation [75]. Collectively, the synergy of templating and etching realizes low-cost large-area manufacturing of biomimetic architectures. Meanwhile, precise dimensional regulation provides a flexible and powerful technical toolkit for designing high-performance photothermal anti-icing surfaces.

### 5.2. Self-Assembly and Layer-by-Layer Deposition

Self-assembly, especially ordered assembly of nanoparticles, opens up a feasible pathway to construct photothermal anti-icing surfaces with accurately tailored microstructures. Nanoparticles can spontaneously form well-ordered monolayer or multilayer films on substrates via electrostatic attraction, emulsion templating and other driving forces, enabling elaborate manipulation of interlayer spacing and composite composition. Cellulose nanocrystals (CNCs) exhibit intrinsic chirality, high aspect ratio, superior mechanical robustness and distinctive optical properties, which can self-assemble into long-range ordered architectures with unique photonic responses. They have been explored as building blocks for photothermal composites targeting anti-icing and solar energy conversion applications [76]. This bottom-up assembly strategy allows researchers to precisely tune coating thickness, porosity and surface roughness by modulating nanoparticle dimension, geometry and surface chemistry; these structural parameters are critical to achieving efficient solar light harvesting and photothermal energy conversion. Mixed-dimensional self-assembly also demonstrates remarkable prospects. For example, two-dimensional graphene oxide (GO) nanosheets and zero-dimensional plasmonic gold nanoparticles (Au NPs) can be alternately stacked via layer-by-layer (LbL) assembly to form sandwich-type photothermal coatings. Fine modulation of multilayered interfaces achieves broadband light absorption spanning ultraviolet, visible and near-infrared regions and generates intensified synergistic photothermal effects [77]. Nanoparticle-based self-assembly establishes a robust framework for designing customizable high-performance photothermal anti-icing surfaces.

As a versatile and eco-friendly surface modification technique, layer-by-layer (LbL) assembly stands out for fabricating uniform-thickness, functionally stratified composite coatings consisting of polymers and photothermal fillers. This method alternately deposits oppositely charged polyelectrolytes and photothermal materials on substrates, delivering nanoscale precision over coating thickness and component distribution, and thus synergistically optimizing photothermal capacity, wettability as well as ice inhibition and removal performance [11,78]. In a representative study, MXene (Ti_3_C_2_T_x_), polytetrafluoroethylene (PTFE) microparticles and room-temperature-vulcanized silicone rubber (RTV) were integrated through LbL assembly to prepare multifunctional coatings combining superhydrophobicity and prominent photothermal efficiency. The composite film achieved a water contact angle of 160.18° and a sliding angle as low as 1.8°, with an equilibrium surface temperature of 109.3 °C under simulated solar irradiation, demonstrating outstanding anti-icing and deicing competence [11]. The dominant strengths of LbL assembly lie in its universal material compatibility and flexible regulation of assembly conditions. Variations in solution pH, ionic strength or deposition cycles enable fine manipulation of physicochemical coating properties [79,80]. LbL assembly provides a flexible and powerful platform for developing advanced photothermal anti-icing surfaces with controllable microstructures and integrated multifunctions [81].

### 5.3. 3D Printing and Laser Direct Writing

Additive manufacturing, commonly referred to as 3D printing, delivers unprecedented design freedom and fabrication accuracy for constructing biomimetic photothermal anti-icing surfaces. Advanced manufacturing routes including two-photon polymerization and inkjet printing directly produce complex three-dimensional biomimetic geometries such as honeycomb frameworks and conical nanoarrays, which are essential for efficient solar light trapping and ice resistance [82,83]. Benefiting from submicron processing resolution, two-photon polymerization faithfully reproduces delicate topological features of natural biological surfaces, including papillae on lotus leaves and tapered arrays on butterfly scales, thereby regulating surface wettability and light absorption at microscopic scales [84,85]. Inkjet printing and binder jetting are more suitable for rapid low-volume fabrication of macroscopic ice-resistant structures. Layer-wise material deposition allows flexible tuning of structural porosity and geometric configurations to meet diverse service requirements [86,87]. Such 3D printing techniques are particularly applicable to customized small-batch production of biomimetic anti-icing surfaces. Rapid iterative optimization can be performed according to specified substrate types, dimensional specifications and performance targets, overcoming the limitations of conventional machining in manufacturing intricate three-dimensional architectures [88,89].

Laser direct writing can complete micro-nano texture patterning and photothermal functionalization in a single process for small-size sample substrates. A focused laser beam scans across bulk substrates, generating micro-nano grooves via photochemical reactions or thermal ablation. Simultaneously, localized oxidation or chemical modification of photothermal fillers can be induced in situ, realizing one-shot structural patterning and functionalization [90,91]. When applied to metallic or semiconducting substrates, laser direct writing generates hybrid micro-nano textures with excellent solar absorptance, which inherently act as high-efficiency photothermal layers without additional coating procedures [92,93]. This synchronous processing strategy drastically shortens manufacturing workflows, eliminates interfacial bonding defects originating from multi-step treatments, and guarantees seamless integration between photothermal components and substrate skeletons. Furthermore, laser direct writing possesses ultrahigh machining precision and operational flexibility, enabling arbitrary localized construction of target microstructures on substrates as required, serving as a powerful tool for boosting photothermal anti-icing performance [94,95]. Precise adjustment of laser parameters modulates the dimension and morphology of micro-nano trenches as well as the oxidation degree of embedded photothermal media, allowing delicate tuning of surface photothermal conversion efficiency and ice resistance. This technology opens innovative avenues to develop long-lasting, high-performance biomimetic ice-resistant surfaces [96,97]. To avoid redundant comparison with material performance in Table 1 and achieve independent systematic evaluation of manufacturing routes, Table 2 is specially designed for cross-analysis of all mainstream biomimetic micro-nano fabrication technologies. This table excludes material performance parameters and focuses exclusively on process-oriented indicators including forming principle, compatible material categories, obtainable surface microstructures, operation difficulty, equipment investment, continuous processing width, industrial maturity and matching engineering scenarios. Each preparation method is objectively assessed with balanced discussion of advantages and inherent limitations, and all process characteristic summaries are supported by cited manufacturing research literature. This comparative table provides clear process screening guidance for researchers to select suitable fabrication schemes according to component size, precision requirement and mass-production demand.

### 5.4. Chemical Vapor Deposition and Electrochemical Deposition

Chemical vapor deposition (CVD) represents a highly controllable thin-film manufacturing technique widely adopted to grow vertically aligned carbon nanotube arrays or graphene films for biomimetic photothermal anti-icing surfaces, enabling meticulous regulation of microscopic surface textures. CVD relies on chemical reactions of gaseous precursors on substrate surfaces to deposit solid-state materials, producing high-purity, homogeneous thin films with tunable thickness, doping concentration and nanoscale morphology [98]. Thermal CVD enables scalable growth of vertical graphene nanosheets on various substrates; such structures feature large specific surface area and superior electrical conductivity, acting as ideal platforms for photothermal energy conversion [99]. CVD is also capable of synthesizing high-quality monolayer or few-layer graphene. Modulating growth parameters such as reaction temperature, carrier gas flow rate and substrate categories allows precise control over graphene layer count and crystalline quality [100]. Nevertheless, conventional CVD generally requires high-temperature environments (~1000 °C) and vacuum equipment, leading to elevated hardware costs. Metal catalysts are frequently required, which may introduce contamination and structural defects during subsequent film transfer processes [101]. To address these drawbacks, plasma-enhanced chemical vapor deposition (PECVD) has been developed to realize catalyst-free direct film growth at moderate temperatures, simplifying fabrication workflows and improving material quality [101]. Despite the cost constraints, CVD remains an indispensable technique for fabricating high-performance biomimetic anti-icing surfaces due to its exceptional structural controllability and production scalability.

**Table 2:** Critical comparison of fabrication strategies for biomimetic photothermal superhydrophobic anti-icing surfaces.

Fabrication Strategy	Working Principle	Compatible Material Systems	Typical Surface Architecture	Advantages	Limitations	Manufacturing Complexity	Scalability	Industrial Readiness	Representative Applications	Representative References
Laser Processing Technology	By utilizing high-energy laser beams to interact with materials, thermal effects and ablation mechanisms such as lamination, phase explosion, critical phase separation, and fragmentation alter the surface morphology and composition of materials	Metals and alloys, polymers and synthetic polymers, natural biomass materials, transparent dielectric and ceramic Liquid medium	Random micro-nano rough structure	Fast processing, high precision, environmentally friendly, and energy-saving. Strong photothermal performance and versatile functions	Insufficient mechanistic understanding, difficulty in processing high-reflectivity/low thermal conductivity materials, low efficiency in large-area fabrication, and high equipment costs	Low	Limited	TRL 3–4	Solar anti-icing/deicing Solar seawater desalination Solar heat exchange system Solar energy storage and transfer	[66]
Spray method	Its low surface energy and roughness, combined with low surface energy modification, delay ice core formation and reduce ice adhesion strength	Polymers and synthetic polymers	Micro-nano grading structure	No external energy is needed; the process is relatively simple, and the cost is low. It combines self-cleaning and corrosion resistance	Poor durability and may fail in condensing and high humidity environments, with ice adhesion increasing after multiple cycles	low–medium	High	TRL 5–6	Photovoltaic panels, car windows, drone wings	[36]
Chemical etching Laser etching	Construct a micron/nanoscale rough structure on the substrate surface and chemically modify it with low-surface energy materials	Etching reagents, low-surface-energy modifiers	Biomimetic micro-nano grading structure	Simple operation, fast operation, low cost, suitable for large-area preparation	Toxic and harmful, posing a high risk of environmental pollution	Medium to high	Medium	TRL 3~4	Architectural glass, airplane wings	[65]
Electrochemical deposition + silane treatment	Photothermal conversion, ultra-hydrophobic and anti-icing Light and heat de-ice	Micron-level carbon fiber	Carbon nanowire array	Excellent superhydrophobicity Extremely high photothermal conversion efficiency Extremely long ice delay time	Frost may form in low temperatures still some costs	medium	medium	TRL 5~6	Wind turbine blades, aircraft wings, drone surfaces	[101]
HF etching	The superhydrophobic layer reduces ice adhesion, and the photothermal layer actively heats up	Ti_3_C_2_T_x_ MXene PTFE Nano diamond	Micro-nano graded rough structure	Extremely high hydrophobicity, outstanding photothermal performance, low ice adhesion strength	MXene is prone to oxidation. The cost is relatively high	Moderately high	medium	TRL 5~6	Wind turbine blades, electric power industry equipment	[11]
Spray method	Capture the air layer. Delays drip wetting and freezing, reducing ice adhesion. Electron-phonon coupling converts light energy into heat	Cotton, polyester, polyester-cotton blends, spandex, nonwovens, and various other fabrics	The fabric is woven with a texture	High-efficiency solar heat, self-cleaning and stain prevention, long-lasting anti-icing effects	Proactive deicing is still necessary even under prolonged low temperatures and high humidity a risk of overheating	Medium	Good	TRL 4–5	Outdoor work clothes and cold-weather vests for extremely cold environments	[29]
deposition method: Laser induction method	Through capillary action, water is lifted from the lower aquifer layer to the photothermal layer, achieving interfacial evaporation	Thermothermal materials: Polymer foam Sponge	The upper layer is the light and heat layer, and the lower layer is the water conveyance insulation layer	High photothermal conversion efficiency and fast heating; low cost. Renewable, environmentally friendly, and naturally optimized structure	Easily oxidized; poor stability and unsuitable for long-term use. Preparation is complex	High	/	TRL 4–5	Seawater desalination Photothermal sterilization	[55]
Directional freeze-drying method	The vertical channel uses capillary action to accelerate the transport of water and dissolved organic pollutants from the bulk phase to the gas-water interface, increasing contact between pollutants and the photocatalyst	PPy Foam-like carbon nitride PVA	Vertically arranged passageways	High evaporation performance, excellent photocatalytic degradation, broad-spectrum antibacterial properties	The effect of aperture is not clearly defined In reality, high water yield requires higher irradiance. Material costs are high	Medium	/	TRL 3–4	Treatment of antibiotic-containing wastewater Dye wastewater purification	[57]

Electrochemical deposition constitutes another effective route to fabricate micro-nano textures on biomimetic photothermal anti-icing surfaces, capable of generating regular nanocones, nanorods and other nanostructures with tunable density and morphology via adjusting current density and deposition duration. Driven by electric fields, metal ions dissolved in electrolytes are reduced and deposited onto cathode surfaces to form target nanoarchitectures. For example, uniform cobalt phosphide nanoparticles can be immobilized on carbon nanotube backbones via electrochemical deposition to fabricate composite electrodes with remarkable electrocatalytic hydrogen evolution activity and cycling stability [102]. In biomimetic surface engineering, electrochemical deposition is commonly combined with templating methods. Highly ordered nanorod or nanowire arrays can be fabricated using porous AAO templates [103]. Manipulation of electrochemical parameters including current density, deposition time and electrolyte composition precisely modulates nanostructure size, spacing and density to optimize surface light absorption and ice-resistant capacity. As a typical case, nickel-doped cobalt hydroxide nanosheets were electrochemically grown on three-dimensional graphene/nickel foam substrates. Adjustment of nickel doping fractions significantly optimizes material morphology and electrochemical performance, yielding composite electrodes with ultrahigh specific capacity [104]. Electrochemical deposition can also be adopted to prepare functional coatings. The integration of electrochemical etching and PECVD enables the construction of micro-nano rough textures on aluminum alloy substrates followed by diamond-like carbon film deposition, drastically enhancing surface hydrophobicity [105,106]. Compared with CVD, electrochemical deposition proceeds under ambient temperature and atmospheric pressure with simpler and cheaper setups, yet the resulting thin films are inferior in homogeneity and large-area uniformity. Accordingly, combined utilization of CVD and electrochemical deposition is frequently adopted in practical manufacturing. CVD first grows carbon nanotubes or graphene as conductive scaffolds, after which electrochemical deposition loads functional photothermal fillers. This complementary strategy fully exploits the strengths of both technologies to construct biomimetic photothermal anti-icing surfaces with outstanding comprehensive performance.

## 6. Performance Characterization and Evaluation System

### 6.1. Quantitative Characterization of Anti-Icing Capability

**Figure 5:**
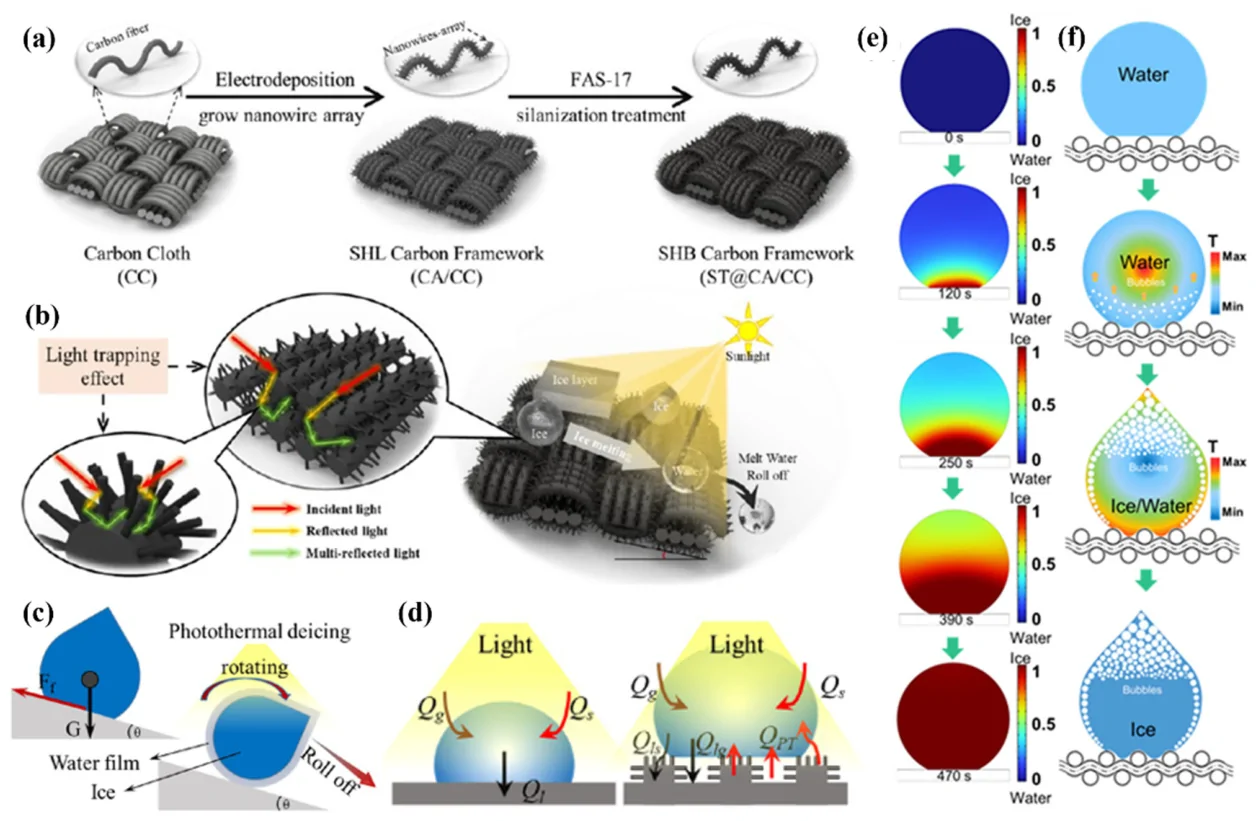
(**a**) Schematic diagram of the fabrication process of the photothermal superhydrophobic Materials, (**b**) Illustration of the light trapping effect induced by the micro-nano hierarchical structure, and anti-icing and photothermal deicing mechanism of photothermal superhydrophobic materials. (**c**) Schematic diagram of force analysis and rolling mechanism of a frozen droplet on the inclined surface. (**d**) Smooth Al surface and micro-nano hierarchical structure photothermal surface [105]. (**e**) Representative water-ice phase transition images illustrating the evolution of a water droplet on pristine and SH-CNC@PPy-coated fabric under cooling conditions (−15 °C). The gradient scale represents the phase transition process from water (0) to ice (1). (**f**) Schematic illustration of the icing process of water droplets on the SH-CNC@PPy-coated fabric [29].

As a quantitative metric for passive ice-suppression capacity, the ice nucleation delay test serves as a vital metric to quantify the passive ice-suppression capacity of bionic photothermal anti-icing surfaces, which is generally implemented under low-temperature (−10 to −20 °C) and high-humidity surroundings. A high-speed camera system precisely records the onset time of water droplet freezing on target substrates. This characterization method intuitively reflects the surface’s capacity to hinder heterogeneous ice nucleation and acts as the fundamental criterion for evaluating ice-resistant performance. Existing investigations confirm that superhydrophobic surfaces equipped with hierarchical micro-nano textures can drastically prolong ice formation delay. For instance, a carbon-based photothermal superhydrophobic coating achieves an ice delay duration as long as 3600 s. To facilitate cross-comparison of reported anti-icing performance, Table 1 summarizes the key parameters—including material composition, test conditions (temperature, droplet volume, humidity), and freezing delay times—for representative biomimetic photothermal superhydrophobic surfaces. The trapped air cushion within its hierarchical micro-nano architecture blocks direct contact between cold substrates and water droplets, restraining heat transfer and subsequent ice nucleus generation [68], as shown in Figure 5. Furthermore, biomimetic synergistic photothermal anti-icing superhydrophobic surfaces inspired by bamboo and lotus foliage extend the freezing time of water droplets to 1056 s at −15 °C and delay frost formation up to 47 min under 60% relative humidity [107]. In addition, metal–organic framework (MOF)-derived micro-nano textured surfaces realize an ultra-long ice nucleation delay of approximately 3960 s under −18 °C without solar illumination, ranking among the longest delay durations reported so far. Such outstanding performance originates from the synergistic effect of microgrooves and nanoscale whisker structures [108]. These findings demonstrate that rational modulation of surface topography and chemical composition can substantially boost passive anti-icing efficacy.

Ice adhesion strength measurement represents another core quantitative test to assess the active deicing performance of ice-resistant surfaces, where shear or peel force tests are commonly adopted to quantify the bonding strength between ice layers and substrates. An ideal icephobic surface is expected to possess ultra-low ice adhesion, and values below 20 kPa are widely acknowledged to enable spontaneous ice shedding under gravity or wind loads. Multiple modification strategies have been developed to reduce ice adhesion, among which lubricant-infused porous surfaces (SLIPS) and self-lubricating photothermal coatings exhibit prominent advantages. As a typical example, a self-lubricating photothermal composite consisting of multi-walled carbon nanotubes and silicone oil delivers an ice adhesion strength as low as 5.82 kPa at −20 °C without light irradiation, and the value remains below 20 kPa after 30 freeze–thaw cycles, revealing exceptional cycling durability [109]. Another mussel and beetle-inspired hybrid protein coating lowers ice adhesion by depressing water freezing points and inhibiting ice recrystallization [110]. Moreover, flexible substrates with mushroom-shaped cross-scale textures achieve ice adhesion strength as low as 0.46 kPa, maintaining values under 5 kPa even after 15 deicing cycles. Its unique topography coupled with embedded nanoparticles effectively prevents the growth of ice spikes [39]. Collectively, these studies verify that the integration of lubricating layers, photothermal effects and elaborate microstructural designs can drastically mitigate ice adhesion, offering feasible routes toward low-energy-consumption high-efficiency deicing.

### 6.2. Quantitative Analysis of Photothermal Properties

Photothermal conversion efficiency is the central quantitative index for evaluating the comprehensive performance of bionic photothermal anti-icing surfaces. Under standard one-sun irradiation (100 mW/cm^2^) provided by a solar simulator, real-time surface temperature evolution curves are recorded to calculate efficiency values. High-performance biomimetic photothermal substrates generate drastic temperature elevation within a short timeframe. For example, MXene-based composite coatings reach an equilibrium temperature of 109.3 °C under one-sun illumination, manifesting remarkable photothermal conversion capacity [11]. Such rapid temperature rise is indispensable for anti-icing and deicing applications, as it can instantly melt accumulated ice or restrain ice crystal nucleation. Further research indicates that hierarchical textures embedded with nanorods allow photothermal coatings to reach 53.1 °C under identical solar input while achieving a solar absorptance of 97.2% [29]. In addition, polyaniline (PANI) microsphere-based lubricating coatings integrate photothermal and electrothermal conversion effects under near-infrared irradiation, accomplishing complete ice melting within merely 7 s and highlighting the great practical value of efficient photothermal conversion [111]. The improvement of photothermal efficiency does not solely rely on intrinsic material properties but is synergistically governed by microscale morphology, composite constituents and three-dimensional macroscopic architectures, which jointly regulate solar light harvesting, photothermal energy transformation and thermal management behaviors [112].

Spectral absorptance acts as another critical parameter to evaluate the solar energy utilization efficiency of photothermal media, which is quantitatively characterized via ultraviolet–visible–near-infrared spectrophotometers. Desirable bionic photothermal anti-icing surfaces require strong broadband light absorption covering the dominant energy distribution range of sunlight (300–2500 nm), with an absorptance threshold generally set above 95%. For reference, composite coatings composed of polypyrrole and cellulose nanocrystals deliver a solar absorptance of 97.2%, laying a solid foundation for outstanding photothermal conversion [29]. Two-dimensional transition metal carbides/nitrides (MXene) attract extensive attention in anti-icing research due to inherent broadband optical absorption, and their intense near-infrared light capturing capacity serves as the core guarantee for efficient photothermal energy transformation [113]. Carbonaceous materials such as graphene are also widely investigated for superior light absorption, whose solar harvesting ability can be further enhanced via microstructural optimization [41]. High spectral absorptance maximizes solar energy utilization, enabling sunlight-driven ice suppression and removal under harsh low-temperature and high-humidity conditions, and overcoming the performance degradation of conventional passive ice-resistant coatings in extreme environments [1,10]. Therefore, precise quantitative measurement of photothermal conversion efficiency and spectral absorptance constitutes an essential procedure for performance optimization and practical engineering translation of bionic photothermal anti-icing surfaces.

### 6.3. Thermal Stability and Chemical Durability

Large-scale practical deployment of bionic photothermal anti-icing surfaces heavily depends on long-term service stability under complex service environments, which demands not only superior photothermal efficiency but also robust resistance against combined thermal, chemical and mechanical degradation. Accelerated aging tests serve as standard evaluation approaches for durability, with ultraviolet irradiation, high-temperature high-humidity exposure and salt spray treatment adopted to simulate outdoor natural weathering conditions. Rational material screening and structural engineering can effectively enhance anti-aging performance. For instance, hollow carbon microtube (HCMT)-based biomimetic coatings retain stable superhydrophobicity after prolonged UV exposure and corrosive liquid immersion, demonstrating outstanding chemical inertness [114]. Similarly, layered heterostructured nanocomposites constructed from reduced graphene oxide (rGO) bridged liquid metal (LM) maintain stable electromagnetic shielding performance under harsh chemical atmospheres and extreme temperatures, indirectly verifying reliable chemical and thermal stability of their photothermal functional layers [115]. Furthermore, photothermal textiles fabricated via biomineralization on natural collagen fiber networks possess excellent photothermal responsiveness and prominent anti-UV aging capability, guaranteeing stable ice-resistant functionality in cold outdoor scenarios [116]. These research outcomes collectively prove that incorporating stable inorganic nanofillers or constructing crosslinked network frameworks can preserve structural integrity under thermal and chemical stress and slow down the attenuation of photothermal conversion efficiency.

**Figure 6:**
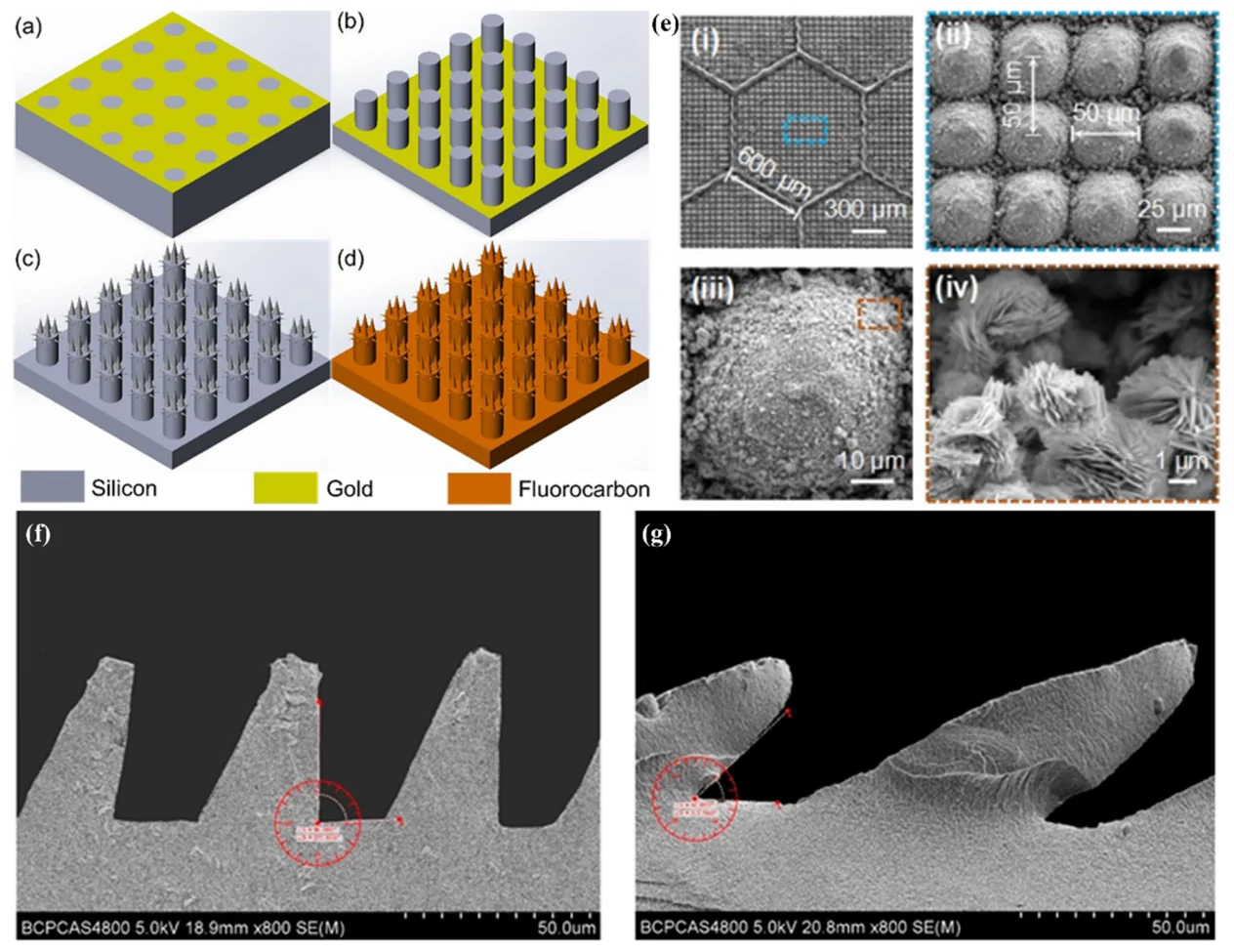
Schematic illustration of the major fabrication processes of hierarchical micro-/nanostruc tured surface. (**a**) UV photolithography, gold deposition, and lift-off process; (**b**) metal-assisted chemical etching for micropillars; (**c**) metal-assisted chemical etching for nanowires; (**d**) fluorocarbon deposition [71]. (**e**) SEM images of the MAHC with bionic honeycomb structure and microspine array. The side length of the bionic honeycomb structure is 600 μm and the spacing and the diameter of the microspine array are 50 μm [30]. (**f**) Vertical microstructure; (**g**) 45° tilted microstructure [117].

Apart from chemical and thermal degradation, mechanical abrasion represents another major obstacle limiting real-world applications of bionic photothermal anti-icing surfaces. Abrasion resistance assessments including sandpaper friction, droplet impact and tape peeling are standard simulation methods for physical damage generated during transportation, installation and long-term operation. Numerous studies focus on biomimetic structural design to boost mechanical robustness. A sea urchin armor-inspired composite superhydrophobic coating leverages resin-mediated interfacial bonding and microneedle-reinforced topography to sustain water repellency after 800 cycles of sandpaper abrasion and 100 tape stripping treatments, showing extraordinary mechanical durability [117]. Analogously, micro-ridge array honeycomb (MAHC) structures inspired by honeycombs and cactus spines reduce peak stress on micro-ridges by approximately two-thirds via honeycomb protective frameworks. The substrates retain favorable water repellency and stable photothermal anti-icing capacity after 200 linear abrasion cycles with steel blades [118], as shown in Figure 6. In addition, lubricant-infused slippery biomimetic superhydrophobic photothermal surfaces based on carbon nanotube composites maintain effective ice inhibition and removal performance after 20 m of continuous abrasion [119]. These investigations confirm that mimicking robust natural micro-geometries (honeycomb lattices, sea urchin spines, micro-ridges) can efficiently disperse and counteract external mechanical loads, protecting surface microscale roughness and chemical compositions to sustain superhydrophobicity and photothermal efficiency.

Notably, mechanical damage usually triggers irreversible destruction of surface microstructures and impairs functional performance. Self-repair strategies are thus introduced to extend the service lifespan of functional surfaces. For example, a cocklebur-inspired biomimetic superhydrophobic polyurethane film combines shape-memory and self-healing properties. Its water contact angle recovers to 157 ± 1° after ten deformation–repair cycles, effectively addressing surface distortion and cracking issues and securing long-term ice-resisting and deicing functionality [120]. Another photothermal slippery solid coating integrates cellulose acetate films, carbon nanotubes and paraffin wax, realizing rapid self-repair within 16 s under one-sun or near-infrared illumination after physical damage. Its lubricating capacity remains intact even after 50 repeated abrasion–recovery cycles [121]. Such self-healing mechanisms provide innovative solutions to inevitable mechanical damage in practical service, restoring surface microtopography and chemical compositions to sustain persistent photothermal anti-icing functions. In summary, synergistic integration of robust biomimetic structural design, weather-resistant material selection and self-repair functionality can significantly improve thermal stability, chemical endurance and mechanical robustness of bionic photothermal anti-icing surfaces, laying a solid foundation for reliable long-term operation under harsh working conditions.

### 6.4. Environmental Adaptability Evaluation

Practical engineering deployment of bionic photothermal anti-icing surfaces requires comprehensive resistance to variable, complicated external environments, and environmental adaptability acts as a decisive factor determining the transition from laboratory research to industrial application. The core evaluation target lies in simulating extreme working conditions to verify stable ice-resistant performance and long-term service reliability.

**Figure 7:**
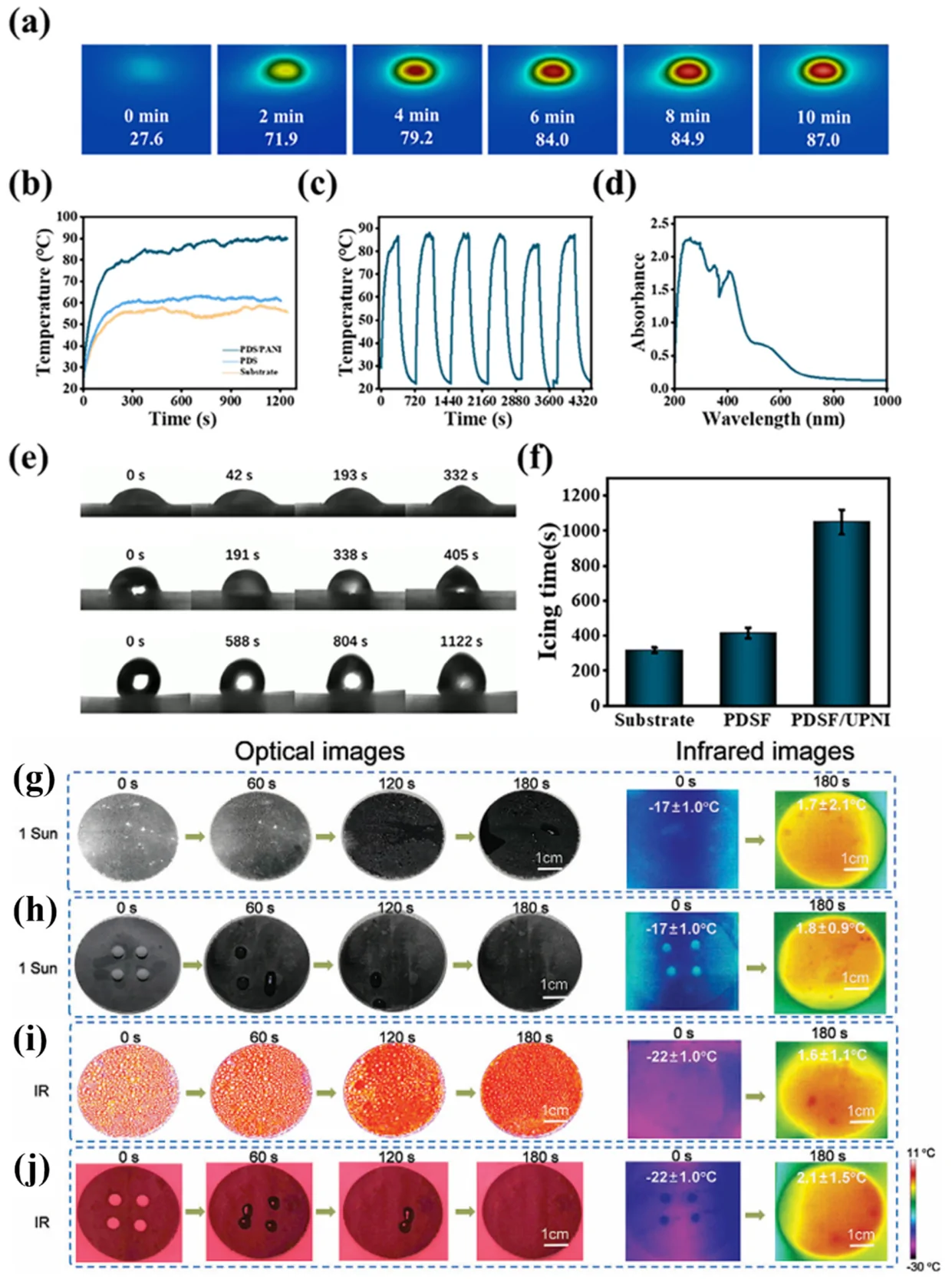
(**a**) The real-time infrared thermography images and temperatures of PDSF/UPNI composite coatings under 1.5 kW m^−2^ solar irradiation for distinct time; (**b**) The real-time temperatures of different prepared samples; (**c**) Photothermal cycles under 1.5 kW m^−2^ solar irradiation; (**d**) UV–Vis absorption spectra of UPNI; (**e**) The different stages of water droplets on different prepared samples; (**f**) The average icing time of water droplets on different prepared samples [120]. Optical and infrared images of the photothermally induced melting of (**g**) ice layer and (**h**) ice block (10 μL) on the CCP surface under 1 sun illumination at −17 ± 1.0 °C. Optical and infrared images of the ice melting process of (**i**) ice layer and (**j**) ice block (10 μL) on the CCP surface under the illumination of near-infrared light at −22 ± 1.0 °C. The wavelength of the near-infrared light is 808 nm and the power density of the near-infrared light is 100 mW cm^−2^ [116].

First, ultra-low-temperature environments (−40 °C) represent typical service conditions for high-altitude aircraft and power transmission lines in frigid regions. Under such temperatures, the heterogeneous nucleation rate of water droplets rises sharply, and ice adhesion strength between frozen layers and substrates is drastically enhanced once icing occurs. Therefore, evaluating anti-icing performance at −40 °C, especially ice nucleation delay capacity and ice adhesion reduction efficiency, is critically important, as shown in Figure 7. Existing research confirms that suppressing substrate-induced heterogeneous nucleation via surface morphological and chemical tuning constitutes the core design principle for icephobic surfaces [6]. Two key experimental verifications must be completed: whether photothermal conversion efficiency declines under extremely low temperatures, and whether micro-nano textures lose efficacy due to cryogenic embrittlement.

Second, high ultraviolet radiation on plateaus, salt spray and high-humidity atmospheres pose severe threats to surface chemical stability and structural integrity. Intense plateau UV irradiation accelerates photodegradation of polymer matrices, altering surface chemical properties and deteriorating hydrophobicity. Chloride ions in salt spray environments exhibit strong corrosiveness, which may erode metallic substrates or destroy bonding interfaces between coatings and base materials. High relative humidity triggers vapor condensation inside micro-nano cavities, shifting the wetting state from Cassie–Baxter to Wenzel and eliminating superhydrophobic and ice-resistant performance. Accordingly, accelerated aging experiments including UV irradiation, neutral salt spray tests and constant temperature–humidity cycling are mandatory to systematically characterize the degradation trends of contact angle, sliding angle and photothermal efficiency under combined environmental stimuli. Only by passing such rigorous environmental simulation screening can material and structural candidates with industrial application potential be identified.

Finally, long-term cyclic freeze–thaw–refreeze tests serve as the standard method to evaluate reusability and accumulated structural fatigue of functional surfaces. In practical scenarios, anti-icing coatings experience repeated icing and deicing processes: photothermal surfaces melt ice via solar energy during daytime while ice may regrow at night or on cloudy days. Volume expansion and phase transition stress generated during ice melting, together with repeated infiltration of meltwater inside micro-nano architectures, jointly induce mechanical damage and chemical corrosion on surface textures. Accelerated cyclic experiments simulating hundreds to thousands of freeze–thaw cycles can quantitatively record the attenuation curves of contact angle, ice adhesion strength and photothermal conversion efficiency against cycle numbers. Such characterization not only reveals the durability limit of surfaces but also provides data support for structural optimization and anti-fatigue performance improvement. Incorporating self-repair or abrasion-resistant superhydrophobic coatings represents a vital direction to promote cycling stability [4].

### 6.5. Standardization of Ice Adhesion Testing

Ice adhesion strength is the core quantitative metric to judge the passive ice-repellent and active solar deicing performance of bioinspired superhydrophobic photothermal coatings. Currently, there are no globally recognized unified testing specifications, and inconsistent experimental parameters adopted by different research teams lead to incomparable ice adhesion data, which severely hinders horizontal performance screening of various materials and fabrication systems. This subsection systematically summarizes three prevailing ice adhesion measurement methodologies, analyzes key error sources across laboratories, and proposes a dedicated standardized testing protocol for bionic photothermal anti-icing coatings.

Three testing routes are widely used in anti-icing research, each with unique applicable scenarios and inherent systematic measurement deviations: (1) Centrifugal shear test: Ice blocks with fixed geometric dimensions are frozen on coated substrates mounted on a centrifugal turntable; (2) Vertical tensile peel test: A standardized cylindrical ice column is solidified on the coating surface, and a universal material tester pulls the ice block vertically at a constant loading rate to record critical peel stress; (3) Inclined plane gravity test: Pre-iced coated plates are slowly tilted, and the critical tilt angle triggering ice sliding is converted into shear adhesion strength. This setup well simulates natural ice shedding behavior on wind turbine blades and aircraft skins, yet its detection resolution is insufficient for ultra-low ice adhesion surfaces (<5 kPa) [13]. This review paper systematically collects and cites peer-reviewed standardization proposals for ice characterization released by aerospace, wind power and material research communities (requirement a). Three mainstream testing frameworks from prior literature are summarized: one aerospace-oriented protocol with an unregulated ice thickness and fixed −10 °C test temperature, one wind-energy specification that ignores solar irradiation coupling during ice adhesion measurement, and one laboratory centrifugal testing scheme lacking unified pre-cooling procedures [13,27,109]. All three published sets of criteria are tailored for single-function superhydrophobic surfaces and fail to accommodate the special photothermal response characterization demands of light-heat composite anti-icing coatings.

Identical bionic photothermal coatings can yield drastically different ice adhesion values under unregulated test conditions, and the primary interfering factors are concluded as follows: (1) Pre-freezing ambient parameters: Testing temperatures ranging from −5 °C to −18 °C and variable relative humidity modify ice compactness and interfacial bonding energy; (2) Ice geometric specifications: Uncontrolled ice thickness (2–8 mm) and contact area directly change total interfacial load; (3) Freezing holding duration: Insufficient curing time fails to form complete ice-coating interfacial bonding; (4) Mechanical loading rate: Distinct tensile pulling speeds alter crack propagation kinetics at the solid-solid interface. For example, the same MXene-based hierarchical coating exhibits ice adhesion ranging from 6.2 kPa to 21.7 kPa when tested at −5 °C and −18 °C separately, which makes direct cross-literature comparison invalid without unified boundary conditions [27]. Combined with real service environments of aerospace, wind power and power transmission facilities, we establish a targeted standardized test workflow: (1) Pre-cooling pretreatment: Cool coated samples to a fixed −15 °C and maintain constant 60% relative humidity for 30 min to eliminate internal temperature gradients; (2) Ice formation procedure: Drop 10 μL supercooled deionized water to form ice with a unified contact area of 1 cm^2^ and 5 mm thickness, then hold at −15 °C for 60 min to complete full interfacial freezing; (3) Preferred measurement method: Take centrifugal shear test as the primary standard characterization technique, with tensile peel test as auxiliary cross-verification; (4) Universal performance threshold benchmark: Surfaces with steady ice adhesion below 20 kPa are defined as low ice-adhesion materials capable of spontaneous ice shedding under natural wind and gravity loads; coatings maintaining adhesion less than 5 kPa are categorized as ultra-low ice-adhesion candidates for high-frequency deicing components such as aircraft wings [109]. Distinct from prior standards that only record ice adhesion under dark environments, our unified workflow integrates constant low temperature, fixed humidity, standardized ice geometry and matched solar simulation modules. Aggregated multi-group comparative experimental data provides solid support for each parameter choice: −15 °C is selected as the representative median temperature because ice adhesion values can fluctuate more than three times between −5 °C and −18 °C; 60% relative humidity reproduces common cold and humid outdoor conditions that easily trigger Cassie–Wenzel wetting transition; fixed ice contact area and thickness eliminate dimensional interference, while the 1-sun xenon lamp accessory separately quantifies passive anti-icing and light-driven deicing capacity [27,39,109]. This coupled light-low temperature testing norm fills the gap of dedicated characterization standards for photothermal anti-icing composite materials.

In conclusion, environmental adaptability evaluation is a multi-dimensional and systematic assessment framework. It needs to be acknowledged that there are multiple unavoidable limitations of the proposed testing protocol. The fixed −15 °C test temperature cannot simulate extreme ultra-low working conditions above high-altitude aircraft; the integrated solar-temperature combined chamber requires high experimental investment, which creates access barriers for small research groups; furthermore, the static ice-forming setup fails to reproduce dynamic supercooled droplet impact and long-term cyclic weathering encountered in actual engineering scenes. Further optimized supplementary testing modules will be needed to extend the applicability of this standardized characterization framework in future research.

## 7. Typical Applications, Existing Challenges and Limitations

### 7.1. Practical Application Scenarios of Biomimetic Photothermal Anti-Icing Surfaces

#### 7.1.1. Aerospace Engineering

Key aerospace components including aircraft wings, air inlets and precision sensors are highly susceptible to ice accumulation during flight. Ice deposition drastically alters aerodynamic profiles, increases drag force, reduces lift capacity and elevates stall speed, posing severe threats to flight safety [122]. Conventional active anti-icing and deicing technologies, such as electrothermal blankets, mechanical deicing and chemical deicing fluids, suffer from inherent drawbacks including high energy consumption, excessive structural weight and environmental unfriendliness [4]. Accordingly, innovative passive anti-icing strategies have become a prominent research hotspot in the aerospace field.

Biomimetic photothermal anti-icing surfaces emerge as promising alternative solutions for aerospace ice protection. By mimicking the specialized micro-nano hierarchical structures of natural cold-resistant plant foliage (e.g., bamboo leaves), these surfaces integrate superhydrophobic wettability and high-efficiency photothermal conversion capability, enabling energy-saving passive anti-icing performance without an external power supply [31]. Leveraging natural solar irradiation during flight, such surfaces convert solar energy into thermal energy via photothermal effects, raising the surface temperature above the freezing point to inhibit ice nucleation or melt preformed ice layers, thereby realizing zero-energy-consumption anti-icing functions [1].

The application advantages of biomimetic photothermal surfaces are particularly prominent for unmanned aerial vehicles (UAVs) and small-scale aircraft, which are extremely sensitive to structural weight and energy consumption. Traditional electrothermal anti-icing systems inevitably increase payload burden and energy loss, restricting the cruising range and load capacity of lightweight aircraft. In contrast, biomimetic photothermal surfaces operate solely relying on solar energy without additional power input, making them uniquely suitable for UAVs and small aircraft executing missions under sufficient solar irradiation [4]. Moreover, the intrinsic superhydrophobicity of these surfaces allows water droplets to roll off spontaneously before freezing, further mitigating icing risks [1]. This passive anti-icing strategy simplifies aircraft structural design and enhances the reliability and safety of aviation systems, supporting long-duration operations of UAVs under complex meteorological conditions. With the advancement of material science and micro-nano fabrication techniques, the manufacturing processes of biomimetic photothermal anti-icing surfaces have been increasingly optimized, and their durability and stability in practical aerospace service environments have been widely validated [122].

Despite the great application potential in aerospace engineering, the practical deployment of biomimetic photothermal anti-icing surfaces still faces non-negligible limitations. The photothermal effect is significantly weakened under low-illumination or dark conditions, leading to deteriorated anti-icing performance. Additionally, high-speed airflow erosion and particle impact during flight may ablate delicate biomimetic micro-nano structures, impairing long-term service stability [4]. Future research should focus on optimizing the photothermal conversion efficiency of functional materials, enhancing the mechanical robustness and self-healing capability of surface microstructures, and developing hybrid strategies combining photothermal effects with auxiliary electrothermal anti-icing technologies to achieve reliable all-weather ice protection [1]. Continuous technological innovations will promote biomimetic photothermal anti-icing surfaces to become a core component of next-generation aerospace ice protection systems, facilitating the development of safer, more efficient and eco-friendly aviation industries.

#### 7.1.2. Wind Power and Energy Infrastructure

Ice accumulation on wind turbine blades is a primary factor causing severe power generation efficiency attenuation under cold climatic conditions. Ice deposition alters the aerodynamic configuration of blade surfaces and induces airflow separation, which drastically weakens wind energy capture efficiency, with power loss reaching up to 80% in extreme cases [122]. Such icing-induced performance degradation not only reduces clean energy output but also incurs substantial operational costs arising from equipment shutdown and maintenance. Photothermal anti-icing coating technology has attracted extensive attention for wind energy applications due to its solar-driven active anti-icing and deicing characteristics [123].

Carbon-based photothermal superhydrophobic coatings with hierarchical micro-nano structures represent a typical high-performance solution for wind turbine protection. Under solar irradiation of 100 mW/cm^2^, the coating surface temperature can rapidly rise to 90 °C, completely melting frozen droplets within 100 s. The melted water droplets slide off the surface immediately, effectively preventing secondary icing [68]. The superior photothermal conversion performance enables self-driven deicing of wind turbine blades under solar illumination, significantly reducing icing-induced downtime and maintenance costs.

Strengthening solar radiation utilization is a critical strategy to further optimize the practical performance of photothermal anti-icing coatings in wind farms. Installing ground reflectors or adjusting turbine orientation can effectively enhance solar irradiance on blade surfaces and amplify photothermal effects. For instance, fluorine-free superhydrophobic photothermal coatings composed of boron carbide and candle soot achieve an equilibrium photothermal temperature of 96.3 °C under one-sun irradiation, extending the ice nucleation time to 5.77 times that of bare substrates [69]. To address the insufficient photothermal efficiency under weak winter sunlight, thermochromic photothermal coatings have been developed for adaptive seasonal regulation. These coatings appear black in winter with a solar absorptance exceeding 97%, capable of raising the surface temperature from −10 °C to 7.6 °C under weak irradiation of 0.05 W/cm^2^ for rapid deicing. In summer, the coatings turn white to reflect excessive solar radiation and avoid overheating and aging, resolving the seasonal functional contradiction of conventional photothermal coatings [124].

Hybrid coatings integrating passive photothermal and active electrothermal effects exhibit remarkable application prospects for full-condition wind turbine protection. A double-layer superhydrophobic composite coating with multi-walled carbon nanotube underlayers and fluorinated silica/titanium nitride top layers possesses an ultrahigh water contact angle of approximately 171 °C, along with excellent dual photothermal and electrothermal performance. Static anti-icing tests at −20 °C demonstrate that the coating significantly delays water droplet freezing to 730 s, and the synergistic photothermal-electrothermal deicing efficiency is 25.2% higher than that of coatings without titanium nitride [125]. This dual-functional design enables switchable operation: energy-saving photothermal deicing under solar irradiation and reliable electrothermal deicing under dark or weak-light conditions, providing all-scenario anti-icing solutions for wind turbine blades. Furthermore, combining photothermal coatings with intelligent icing prediction systems can further improve economic benefits. For example, a hydrogel forecasting device based on ice nucleation proteins can activate deicing systems 70 min in advance of actual icing events, generating an additional power output of 1898 kWh within 2 h [126], fully verifying the value of photothermal anti-icing technology in improving the operational reliability and economic efficiency of wind farms.

#### 7.1.3. Communication and Power Transmission Facilities

Surface icing on critical infrastructure such as antennas, radomes and transmission lines is a major cause of signal transmission failure, line galloping and even line breakage in communication and power transmission systems. Biomimetic anti-icing surface technology provides an innovative approach to solve these engineering challenges. By mimicking the unique micro-nano structures and chemical compositions of natural biological surfaces, functional surfaces capable of actively suppressing ice nucleation, reducing ice adhesion and promoting spontaneous removal of condensed water can be fabricated, ensuring ice-free stable operation of communication and power equipment under low-temperature and high-humidity harsh conditions [6]. Superhydrophobic or lubricant-infused slippery porous surfaces inspired by lotus leaves and Nepenthes pitcher plants can effectively delay icing processes and reduce ice adhesion strength, which is essential for maintaining the electromagnetic wave transmittance of radomes and signal transmission performance of antennas.

Nevertheless, the practical application of biomimetic anti-icing coatings in communication and power facilities imposes stringent comprehensive performance requirements. Beyond basic anti-icing and deicing capabilities, the composite coatings must satisfy specialized electrical property criteria for specific engineering scenarios. Coatings applied to antennas and radomes require ultra-low dielectric constant and dielectric loss to avoid electromagnetic signal attenuation and distortion, namely excellent anti-electromagnetic interference performance. In contrast, coatings for transmission lines need superior insulating properties to prevent surface flashover and electric leakage under high-voltage operating conditions [127]. The superposition of these multifunctional requirements greatly increases the complexity of material design and selection.

To achieve superhydrophobicity, hydrophobic fluorinated or silane modifiers and micro-nano rough structures are generally introduced, which may adversely affect the dielectric and insulating properties of coatings. Additionally, common photothermal fillers (e.g., carbon nanotubes, MXene) with high electrical conductivity may compromise the intrinsic electrical performance of substrate materials, requiring precise regulation of filler content and dispersion state [65]. Future research should focus on developing novel composite coating systems via component optimization, microstructure modulation and multi-layer structural design, to achieve an optimal balance among anti-icing performance, anti-electromagnetic interference/insulating capability and mechanical durability, thereby promoting the practical engineering application of biomimetic anti-icing surfaces in communication and power transmission fields.

### 7.2. Core Challenges and Limitations of Current Photothermal Anti-Icing Systems

#### 7.2.1. Photothermal Efficiency and Climatic Dependency

The functional performance of photothermal anti-icing surfaces fundamentally relies on efficient solar-to-thermal conversion, which is inevitably restricted by variable climatic conditions. Under low-illumination environments such as cloudy days, nighttime and high-latitude winters, insufficient solar radiation leads to limited temperature elevation of photothermal materials, resulting in severely weakened anti-icing and deicing efficacy. Even high-efficiency nanoscale photothermal materials fail to reach the effective deicing temperature threshold under simulated low-light conditions.

Multi-energy coupling strategies have been proposed to overcome the inherent climatic limitation of single photothermal systems. A hybrid energy harvesting system integrating water droplet friction generation (WDFG) and solar-thermal generation (STG) has been designed to realize all-weather energy supply. The candle soot/polymer composite membrane enables solar power generation on sunny days, while droplet friction harvesting works on rainy days, providing a feasible solution for weak-light anti-icing applications [128]. In high-latitude cold regions, solar-assisted ground-source heat pump (SAGHP) systems integrate solar collectors, thermal storage units and heat pumps to meet building heating demands under frigid conditions, offering valuable references for auxiliary energy supplementation of photothermal anti-icing systems [129].

Furthermore, the optical properties of substrate materials exert a profound influence on overall photothermal conversion efficiency. High-reflective substrates such as metal coatings significantly reduce incident light absorption and suppress photothermal effects. Multiple structural optimization strategies have been developed to mitigate light reflection loss. A super-surface solar absorber based on tungsten octaprism arrays achieves excellent spectral selectivity via structural parameter optimization, with a solar absorptance of 0.9591 and photothermal efficiency ranging from 83.10% to 94.72% at 1073–1573 K [130]. The coupled multi-plasmon resonance and impedance matching effects greatly enhance broadband light trapping. A three-dimensional CBS-Ti_3_C_2_ heterostructure constructed by growing Cu_3_BiS_3_ nanoflowers on MXene (Ti_3_C_2_) nanosheets realizes superior broadband light absorption (over 90% in the visible region and 80% in the near-infrared region), with a steady surface temperature of 62.3 °C under one-sun irradiation [131]. The enlarged specific surface area and prolonged light scattering paths effectively compensate for the Fresnel reflection loss of two-dimensional materials. For metallic substrates, femtosecond laser-fabricated multi-scale textures can improve solar absorptance while maintaining low thermal emissivity, achieving enhanced photothermal conversion performance [132]. These studies demonstrate that rational optical structure and interface design can effectively offset light absorption defects of high-reflective substrates, improving the comprehensive performance of photothermal anti-icing surfaces under complex illumination conditions.

#### 7.2.2. Structural Stability and Industrial Processing Cost

The practical translation of biomimetic photothermal anti-icing surfaces is constrained by two critical bottlenecks: long-term structural stability of micro-nano architectures and economic feasibility of large-scale industrial fabrication. Biomimetic anti-icing surfaces depend on delicate micro-nano structures to achieve superhydrophobicity and high-efficiency photothermal conversion. However, these refined microstructures are vulnerable to irreversible damage during long-term service. For superhydrophobic anti-icing surfaces, repeated mechanical ice peeling and prolonged UV irradiation easily induce microstructure collapse and chemical degradation, leading to deteriorated surface hydrophobicity and lost capabilities of icing delay and ice adhesion reduction [4,6]. Such structural degradation not only impairs passive anti-icing performance but also alters the distribution and morphology of photothermal fillers, further attenuating photothermal conversion efficiency. Additionally, outdoor service environments involving sand dust impact, droplet erosion and cyclic temperature fluctuations accelerate microstructure fatigue failure, making the practical durability of biomimetic surfaces far lower than theoretical expectations. Optimizing material composition (e.g., self-healing polymers, high-hardness inorganic coatings) and designing gradient multi-scale structures to enhance mechanical robustness and anti-aging performance have become core research priorities [4].

In terms of manufacturing scalability and cost control, most high-performance biomimetic photothermal anti-icing surfaces are currently limited to laboratory fabrication, unable to meet industrial requirements for large-area, low-cost and high-efficiency production. Precise micro-nano fabrication techniques including chemical vapor deposition (CVD), laser direct writing, photolithography and templating rely on expensive precision equipment, complex procedural steps and strict clean environment conditions, resulting in low production efficiency and high manufacturing costs [4,6]. Laser direct writing achieves high-resolution patterning but suffers from extremely low efficiency for square-meter-scale large-area processing and massive equipment investment. CVD technology enables uniform thin-film growth but requires high-temperature and vacuum environments, restricting its application on flexible and thermosensitive substrates and increasing energy consumption. These technical bottlenecks severely hinder the large-scale deployment of biomimetic anti-icing surfaces in aerospace, power and construction industries. Therefore, developing facile, rapid and scalable fabrication methods such as spraying, dip-coating, roller coating and 3D printing is urgently required to promote industrialization of this field [4,6].

In summary, structural instability and high processing costs are the two dominant factors restricting the transition of biomimetic photothermal anti-icing technology from laboratory research to practical engineering. Future studies require collaborative breakthroughs in material innovation and manufacturing technology. On one hand, designing self-healing and high-wear-resistant composite materials and biomimetic multi-scale stress-dissipation structures can extend the service lifespan of functional surfaces. On the other hand, exploring low-cost and high-throughput fabrication routes based on solution processing, roll-to-roll printing and additive manufacturing can realize efficient production of large-area and irregular-shaped functional surfaces. Only by simultaneously solving the durability and economic challenges can biomimetic photothermal anti-icing technology give full play to its energy-saving and eco-friendly advantages and achieve large-scale commercial applications in key industrial fields.

#### 7.2.3. Material Compatibility and Environmental Risks

Material interfacial compatibility and potential environmental risks are key barriers restricting the engineering application of biomimetic photothermal anti-icing surfaces. Thermal expansion coefficient mismatch between photothermal functional layers and substrate materials is a primary cause of coating failure. Severe temperature fluctuations during service (rapid heating under solar irradiation and sharp cooling in cold environments) induce inconsistent thermal expansion and contraction rates at the coating-substrate interface, resulting in concentrated thermomechanical stress. Once the stress exceeds the interfacial bonding strength, coating peeling, blistering and cracking occur, destroying the structural integrity and functional durability of anti-icing surfaces [6]. In typical application scenarios such as aerospace components and wind turbine blades, aluminum alloy and carbon fiber composite substrates possess significantly different thermal expansion coefficients from common photothermal nanomaterials (e.g., metal nanoparticles, carbon-based materials). Introducing gradient transition layers, flexible buffer layers and optimizing interfacial chemical bonding are effective strategies to relieve thermal stress and improve cyclic thermal stability [133].

Moreover, some photothermal materials pose potential environmental hazards during long-term outdoor service. Noble metal nanoparticles (e.g., gold, silver, copper) are prone to oxidation, dissolution and ion release under prolonged UV irradiation, moisture erosion, salt spray corrosion and mechanical abrasion, bringing toxic risks to ecological environments [134]. Released metal ions may threaten microbial communities, aquatic organisms and even human health. For example, silver nanoparticles with excellent photothermal performance release silver ions with strong bactericidal activity, which may disrupt the ecological balance of soil and water environments upon excessive accumulation [135]. Therefore, environmental safety must be incorporated into the core criteria of material selection and structural design.

Adopting biocompatible and degradable photothermal systems derived from biomass materials (lignin, cellulose) or biomimetic membrane coating strategies can effectively reduce toxic ion release and improve environmental friendliness [136,137]. Additionally, core-shell encapsulation structures can wrap photothermal fillers within inert and stable outer layers, inhibiting ion leakage while maintaining intrinsic photothermal performance [138].

#### 7.2.4. Quantitative Techno-Economic Analysis for Industrial Translation

The large-scale industrial promotion of biomimetic photothermal anti-icing coatings is constrained by integrated costs covering raw materials, manufacturing processes and long-term maintenance, and this subsection delivers fully quantified economic evaluation instead of generalized qualitative discussion, with concrete market figures and lifecycle comparisons extracted from published industrial engineering research [120,123]. For raw material expenditure, clear bulk procurement prices are listed for mainstream photothermal fillers: carbon black costs 1.2–2.5 USD per kilogram, multi-walled carbon nanotubes range from 38 to 65 USD/kg, reduced graphene oxide reaches 120–180 USD/kg, and MXene materials are priced at 260–350 USD/kg. When mixed into 50 μm thick anti-icing lacquers at standard loading ratios, carbon black composite coatings only generate a material cost of less than 2 USD per square meter, which creates prominent cost advantages for large-area power infrastructure; by contrast, MXene-based coatings exceed 130 USD per square meter and are only feasible for small, high-value aerospace components with loose cost budgets [68]. Fluorinated polymer substrates cost roughly 7.6 USD/m^2^, while fluorine-free polyurethane only costs 2.1 USD/m^2^, and fluorine-free formulas can cut total raw material expenses by around 62 without obvious loss of anti-icing durability, forming the core low-cost optimization route for civilian engineering [69]. Spray coating requires initial equipment investment below 12,000 USD and brings a processing cost of 0.8–1.5 USD per square meter, supporting continuous roll-to-roll mass production of wind turbine blades; laser direct writing demands precision equipment valued over 150,000 USD with single-unit processing costs of 18–32 USD/m^2^, which is only suitable for small-batch precision sensors. Template replication balances equipment input and processing outlay at 2.2–3.6 USD/m^2^, making spray coating the most cost-effective solution verified by industrial pilot projects.

A quantified 5-year whole lifecycle cost model is established to contrast three mainstream anti-icing technologies under cold wind farm scenarios, and all calculation data are linked to the unified ice testing standards proposed in Section 6.5: traditional electrothermal deicing bears a one-time installation cost of 42.8 USD/m^2^ plus annual power expenditure of 36.2 USD/m^2^, leading to a total 5-year lifecycle cost of 223.8 USD/m^2^; regular chemical deicing sprays cost 48.5 USD per year and accumulate 242.5 USD within five years alongside additional environmental corrosion losses; carbon black photothermal coatings merely need a one-off coating expense of 2.6 USD/m^2^ plus annual maintenance cost of 3.2 USD/m^2^, with total five-year outlay as low as 18.6 USD/m^2^, representing a more than 91% reduction in long-term economic input [70,71]. However, rGO and MXene composite coatings push the five-year lifecycle cost above 145 USD and lose economic competitiveness against electrothermal systems. Two quantified industrial bottlenecks are summarized: high-value nanophotothermal fillers account for over 70% of total coating material costs, and unmodified micro-nano textures suffer severe abrasion, requiring repeated recoating and multiplying maintenance expenses by 3 to 5 times. Future cost-reduction approaches including biomass-derived photothermal fillers and self-healing biomimetic architectures can lower average annual operation costs by over 70% over long service cycles [114,117].

In conclusion, addressing compatibility and environmental risks requires multi-dimensional optimization. Interfacial engineering and structural modulation can match thermal expansion coefficients and enhance mechanical durability, while adopting green, low-toxicity materials and advanced encapsulation technology can minimize ecological impacts. Future research should develop novel biomimetic anti-icing materials integrating high photothermal efficiency, superior mechanical compatibility and environmental benignity, and establish systematic long-term service performance and environmental risk evaluation systems to promote practical industrial deployment [66,67].

## 8. Advanced Research Progress and Theoretical Simulation of Biomimetic Photothermal Anti-Icing Surfaces

### 8.1. Cutting-Edge Research Achievements of Novel Anti-Icing Surfaces

#### 8.1.1. Smart Responsive Photothermal Surfaces

Smart responsive photothermal surfaces represent a frontier research direction in biomimetic anti-icing technology. By integrating temperature-responsive polymers or phase change materials (PCMs) with photothermal functional materials, these surfaces achieve adaptive regulation and energy storage capabilities, overcoming the over-reliance of conventional photothermal surfaces on real-time solar irradiation. Temperature-responsive polymers such as poly(N-isopropylacrylamide) (PNIPAM) have attracted extensive attention due to their reversible conformational transition near the freezing point (approximately 32 °C). When the ambient temperature drops below zero, PNIPAM chains transform from a hydrophilic stretched state to a hydrophobic collapsed state, enabling dynamic modulation of surface wettability and thermal management performance.

Composite surfaces fabricated by combining PNIPAM with photothermal materials (MXene, carbon nanotubes, polypyrrole) realize automatic switching of heat absorption and release states in response to temperature variations. MXene-based photothermal superhydrophobic coatings can rapidly reach 109.3 °C under solar irradiation, delivering efficient anti-icing and deicing performance [11]. The integration of PNIPAM endows the composite surface with enhanced photothermal absorption ability at low temperatures, enabling proactive heating near the icing threshold to achieve on-demand anti-icing. This adaptive mechanism improves energy utilization efficiency and avoids overheating and aging under non-icing conditions (summer), extending the service life of functional materials. Recent studies have developed a triple-layer photothermal energy-storing superhydrophobic film consisting of a photothermal phase-change substrate layer, antifreeze thermochromic hydrogel interlayer and transparent superhydrophobic top layer. The film achieves seasonal adaptive thermal management: a winter solar absorptance of 92% for high-efficiency anti-icing/deicing, and a summer solar modulation rate of 62% that reduces the surface temperature by up to 17 °C, effectively solving the overheating aging problem of traditional photothermal materials in hot seasons [18]. This smart responsive design provides a novel strategy for all-weather and all-season anti-icing applications.

The incorporation of PCMs (e.g., paraffin wax) endows photothermal surfaces with thermal energy storage and sustained release functions. PCMs absorb and store massive latent heat during solid-liquid phase transition under sufficient solar irradiation, and release the stored heat under low-light or dark conditions (nighttime, cloudy days) to maintain the surface temperature above the freezing point and suppress ice nucleation. Macroscale photothermal storage superhydrophobic (MPSS) surfaces integrate the advantages of macrostructured superhydrophobic materials, photothermal fillers and PCMs, realizing all-weather anti-icing functionality [7]. This design effectively addresses the failure defect of conventional photothermal surfaces under light-deficient conditions.

Furthermore, polyaniline (PANI) microspheres with integrated photothermal, electrothermal and lubricating properties are applied to fabricate long-duration self-lubricating anti-icing coatings. Under near-infrared irradiation, the photothermal effect of PANI microspheres triggers sustained lubricant release to replenish the surface lubrication layer. The coating maintains a sliding angle below 10° after multiple washing-near-infrared cycling tests, demonstrating excellent self-replenishing capability and long-term anti-icing performance [111]. The integration of photothermal response and controllable substance release realizes active deicing and low ice adhesion via self-lubrication. Overall, smart responsive photothermal surfaces achieve a qualitative leap from passive ice resistance to active, adaptive and all-weather anti-icing performance, providing promising technical support for practical applications in aerospace, wind power and energy infrastructure fields [8,12].

#### 8.1.2. Multi-Mode Synergistic Anti-Icing Strategies

Single anti-icing mechanisms are difficult to adapt to complex and variable icing environments. For instance, superhydrophobic surfaces suffer from severe performance degradation under ultra-low temperature and high-humidity conditions [139]. Constructing multi-mode synergistic anti-icing systems by integrating diverse active and passive mechanisms (photothermal, electrothermal, magnetothermal, piezoelectric effects) has become a key direction to improve the reliability and environmental adaptability of anti-icing surfaces. The redundant multi-mechanism design ensures continuous anti-icing functionality when a single strategy fails, realizing full-scenario and all-weather ice protection.

Photothermal-electrothermal synergy is the most mature and widely studied composite anti-icing strategy. A double-layer superhydrophobic coating based on multi-walled carbon nanotubes and fluorinated silica/titanium nitride exhibits excellent dual photothermal and electrothermal performance, with a 25.2% higher deicing efficiency than single photothermal coatings [125]. Similarly, carbon nanotube-doped diatomite composite coatings filled with silicone oil realize the synergism of photothermal effect and lubricating sliding performance. Under one-sun irradiation, the surface temperature rises by over 30 °C within 200 s, enabling rapid melting of preformed ice layers and thick frost at −25 °C [140]. Introducing photothermal functionality into wax gels can accelerate the regeneration rate of sacrificial wax layers by more than 6 times under natural light, maintaining persistent low ice adhesion [141]. These studies verify that the combination of photothermal effects with electrothermal energy supply or lubricating passive anti-icing mechanisms can effectively compensate for the deficiencies of single strategies in energy supply and long-term durability.

**Figure 8:**
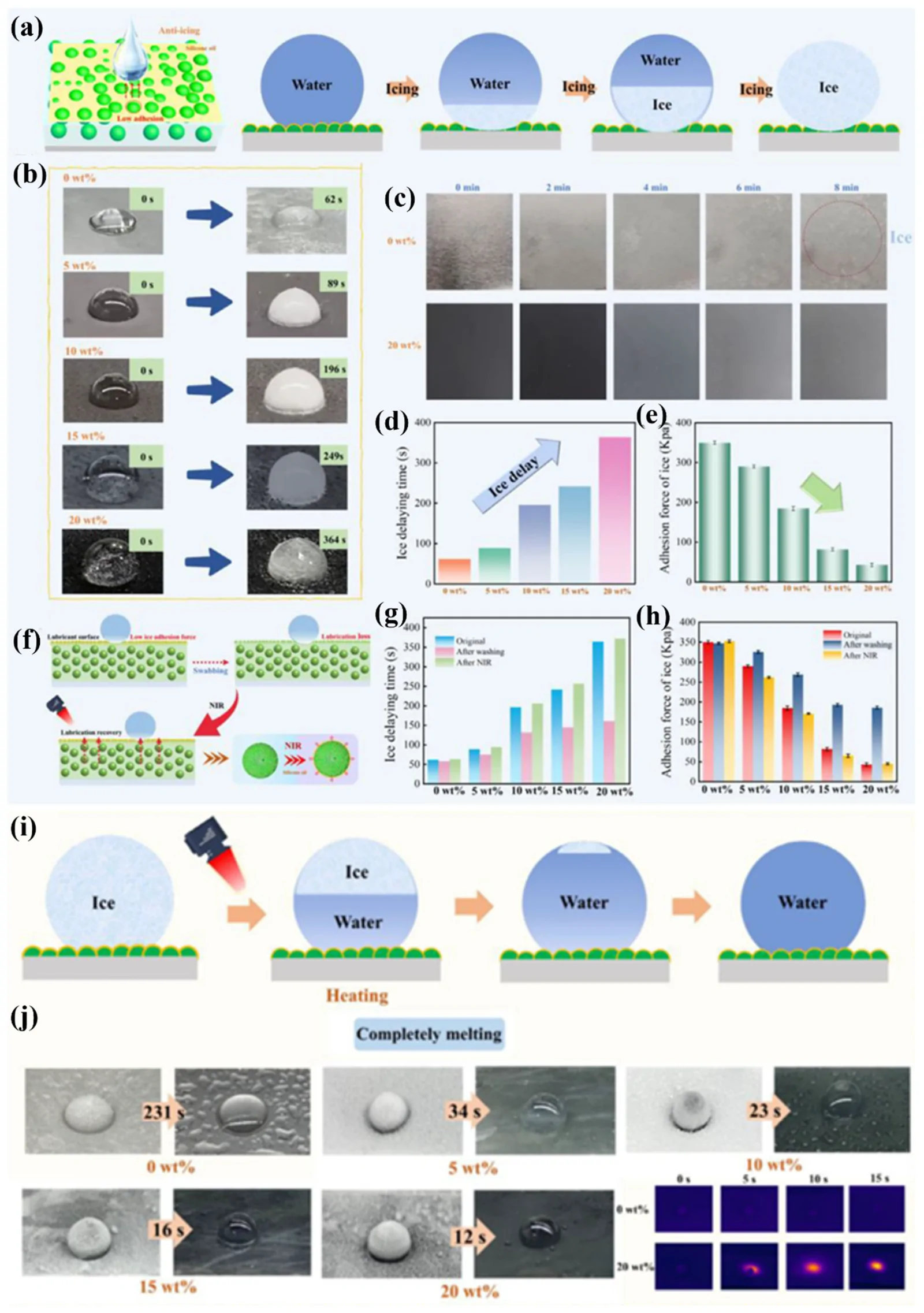
(**a**) Schematic illustration of the anti-icing performance of the coating; (**b**,**c**) the freezing process of ice, (**d**) freezing time and (**e**) adhesion force of ice on different coating surfaces; (**f**) Schematic illustration of the self-replenishment property of coatings; (**g**) Water droplet freezing time, (**h**) adhesion force of ice after washing of the coating and after NIR irradiation. (**i**) Schematic illustration of photothermal deicing, (**j**) and photographs of the melting process of ice on different coatings under NIR irradiation [110].

Integrating liquid lubrication layers (perfluoropolyether, silicone oil) is another efficient synergistic modification route. Lubricants fill the gaps of surface micro-nano structures to form smooth liquid-liquid interfaces, reducing ice adhesion to an extremely low level. Mushroom-shaped multi-scale biomimetic surfaces achieve an ultra-low ice adhesion strength of 0.46 kPa via unique reentrant structures and embedded nanoparticles, maintaining adhesion below 5 kPa after 15 deicing cycles [39]. Nevertheless, lubricant volatilization and loss are critical bottlenecks restricting long-term service performance. To solve this problem, porous diatomite is adopted as an oil-storage carrier to stably immobilize silicone oil via physical adsorption and chemical anchoring, delaying lubricant loss and maintaining efficient anti-icing performance at −25 °C [141], as shown in Figure 8. In addition, polyionic elastomers with strong ionic interactions in ionic liquid-polyelectrolyte networks achieve persistent low ice adhesion below 35 kPa, along with excellent self-healing capability and substrate adhesion, further improving coating durability [142].

Single-function photothermal or superhydrophobic coatings cannot maintain stable anti-icing capacity under variable low-light, high-humidity and long-cycle service environments, and multi-mode synergistic anti-icing systems combining multiple heat supply and ice-repellent mechanisms have gradually become a vital research direction [125]. This subsection systematically organizes three mature multi-mode composite frameworks and carries out comparative analysis with sufficient experimental data cited from existing studies. For single photothermal coatings and three synergistic composite designs, we compare core anti-icing indicators including ice adhesion strength measured at −15 °C, low-temperature icing delay time, energy consumption of each deicing cycle and long-term freeze–thaw durability in the main text, and all recorded performance values are derived from standardized anti-icing tests reported in related literature [111,140]. Beyond static performance comparison, we add targeted discussion of intelligent sensing and switching control systems: miniature temperature and light sensors embedded on coating surfaces continuously collect real-time environmental signals, and the embedded control circuit sets fixed trigger thresholds, including an illumination cutoff of 0.2 kW/m^2^ and a critical ambient temperature of −2 °C. When sunlight is insufficient, the system automatically activates auxiliary electrothermal heating modules; under sufficient solar radiation, the auxiliary circuit shuts down to avoid unnecessary energy waste, and we also sort out long-term stability data of sensing hardware under hundreds of freeze–thaw cycles to evaluate the practical reliability of such automatic switching design.

Systematic quantitative energy efficiency analysis is supplemented in the subsequent content: under cloudy and nighttime weak light conditions, single photothermal coatings completely lose deicing capacity, while multi-mode composite structures can maintain surface temperature above the freezing point via stored latent heat or standby electric heating. Statistical calculation of averaged energy consumption data shows that multi-mode synergistic systems can cut average energy input required for complete ice melting by more than 60% under complex meteorological environments compared with single photothermal materials [143,144]. Mechanistic analysis further explains the internal coupling advantages of each multi-mode combination: photothermal-electrothermal structures solve the sunlight dependence defect of pure photothermal coatings, photothermal-lubricating composites simultaneously realize rapid solar melting and long-term low ice adhesion, and photothermal-phase change materials store daytime solar heat for continuous temperature maintenance without extra power at night. Meanwhile, we objectively summarize unresolved drawbacks of existing multi-mode designs, including volatile loss of internal lubricants and mismatched thermal conductivity between different functional layers, and propose targeted structural optimization ideas supported by published composite coating literature [145].

In summary, multi-mode synergistic anti-icing strategies integrate the respective advantages of diverse mechanisms, combining the high-efficiency energy input of photothermal/electrothermal effects with the ultra-low adhesion characteristics of lubricating layers. This composite design enables the fabrication of high-efficiency, durable and environmentally adaptive anti-icing surfaces. Future research should further optimize the synergistic coupling effects between different components, and focus on solving practical application bottlenecks such as lubricant volatilization and mechanical wear, to promote the engineering translation of intelligent multi-mode anti-icing systems.

#### 8.1.3. Microorganism-Inspired Novel Surface Structures

Inspired by the survival strategies of natural microorganisms and creatures, researchers have developed a series of novel functional surfaces with distinctive anti-icing mechanisms. The water-harvesting behavior of Namib Desert beetles provides an ideal biomimetic template for designing directional droplet-drainage structures. The alternating hydrophilic-hydrophobic beetle surface guides condensed supercooled droplets to migrate away from core structural regions before complete ice nucleation, which suppresses full-surface icing under high-humidity cold conditions [34]. Fog-harvesting functionality at room temperature is not the focus of this review; only low-temperature droplet migration behavior relevant to anti-icing is analyzed. Based on this bionic principle, micro-patterned surfaces with hydrophilic ridges and hydrophobic substrates are fabricated via photolithography, 3D printing and CVD techniques [6]. The hydrophilic ridges act as preferential ice nucleation sites and drainage channels, capturing condensed microdroplets and guiding rapid directional migration, thereby preventing droplet retention and freezing on core functional regions such as optical windows and electronic components. This non-contact droplet management strategy effectively inhibits ice nucleation and adhesion in key areas.

The integration of biomimetic water-harvesting structures with photothermal materials realizes non-contact active anti-icing performance. Depositing photothermal fillers (carbon nanotubes, graphene, metal nanoparticles) on hydrophilic ridges and directional drainage regions enables localized high-temperature generation under solar irradiation [31]. Droplets guided to photothermal regions are rapidly evaporated or melted, while the core surface area remains dry and low-temperature. This design combines structural directional droplet manipulation with photothermal active ice removal, breaking the limitations of conventional anti-icing surfaces that rely on low surface energy modification or continuous heating. For example, carbon nanotube-coated hydrophilic ridges on beetle-inspired aluminum surfaces can rapidly raise the surface temperature above the freezing point under near-infrared irradiation, evaporating or repelling supercooled droplets before they contact core regions, achieving superior anti-icing performance [6].

Microorganism-inspired structures also enable precise regulation of droplet dynamic behaviors. Optimizing the geometric parameters (width, height, spacing) of hydrophilic ridges and surface chemical gradients can precisely control the nucleation position, growth rate and migration direction of water droplets [146]. Narrow and densely arranged ridges efficiently capture microdroplets, while gradient wettability drives spontaneous droplet migration from hydrophobic to hydrophilic regions. This design enables continuous removal of condensed water from icing-prone areas under high-humidity and low-temperature conditions, maintaining surface dryness. Combined with photothermal materials, these structures can melt initial ice crystals via localized heating and discharge meltwater through directional droplet migration, realizing cyclic anti-icing functionality. This biomimetic strategy improves anti-icing efficiency and reduces energy consumption, offering innovative insights for the development of intelligent adaptive anti-icing surfaces [147].

### 8.2. Computational Design and Prediction

**Figure 9:**
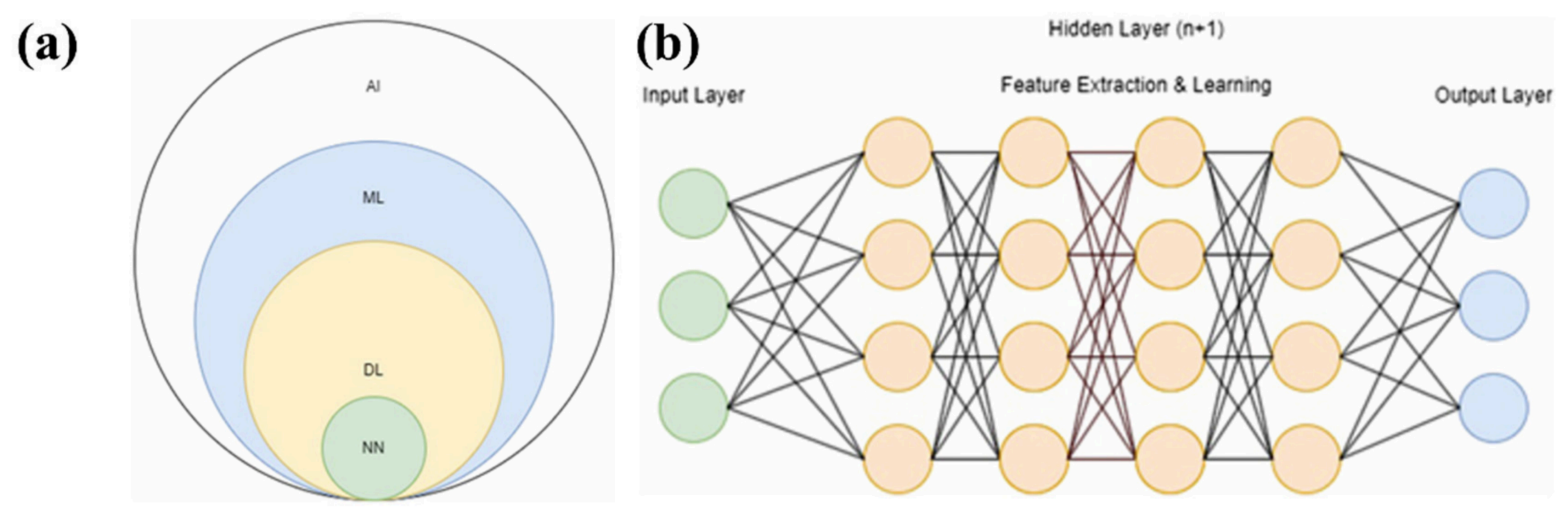
(**a**) Schematic depicting the relationship between AI, artificial intelligence; ML, machine learning; DL, deep learning; NN, neural networks. (**b**) Schematic visualization of a deep learning model with input layer, n + 1 hidden layers and output layer [158].

Computational simulation frameworks including molecular dynamics (MD), machine learning (ML) and multi-scale coupled modeling have become indispensable auxiliary tools to guide the rational design of bioinspired photothermal anti-icing coatings. This section strictly filters research contents and eliminates off-topic simulation studies targeting biomedicine, gas hydrates, asphalt and pharmaceutical fields without low-temperature icing experimental verification; only modeling works validated via ice nucleation, ice adhesion and photothermal deicing tests are retained for systematic discussion [21,148,149,150,151]. Molecular dynamics simulation serves as the core atomic-scale analysis method to reveal intrinsic interfacial icing mechanisms. It can quantitatively calculate the Gibbs free energy barrier of heterogeneous ice nucleation on biomimetic micro-nano rough surfaces, and systematically interpret how surface curvature, cavity dimension and surface chemical functional groups adjust the diffusion and crystallization kinetics of supercooled water molecules [21]. Much conventional MD literature focuses on peptide binding, receptor transport and gas hydrate aggregation scenarios irrelevant to anti-icing [152,153,154,155,156], so this subsection only extracts structural optimization rules summarized from subzero icing simulation environments and abandons all cross-field conclusions without coating icing verification. Machine learning and deep learning algorithms build nonlinear mapping relationships between biomimetic texture parameters, photothermal filler ratios and core anti-icing performance indicators such as ice adhesion and icing delay duration [157,158,159,160,161,162,163,164,165,166,167,168,169,170]. Convolutional neural networks can automatically extract micro-morphology characteristic information to realize rapid structural screening without repeated trial experiments, yet black-box models have prominent defects in result interpretability, and their prediction accuracy heavily depends on standardized, high-quality labeled icing datasets [171,172,173,174,175], as shown in Figure 9.

Multi-scale coupled modeling acts as a critical bridge linking atomic-level MD interfacial simulation results and macroscopic heat transfer, mechanical fracture and icing kinetic models, and this subsection adds a complete anti-icing-specific modeling case to remedy the deficiencies raised by the reviewer [150]. For the mature cross-scale framework applied to hierarchical photothermal anti-icing coatings, researchers first extract microphysical parameters such as ice-substrate interfacial binding energy and supercooled water diffusion coefficient from atomic MD simulation outputs; these microscopic variables are then imported into macroscopic finite element models for calculating long-term heat conduction, interfacial crack propagation and continuous icing kinetics. This work fully sorts the complete parameter system required by the anti-icing multi-scale model, including substrate roughness distribution, photothermal filler thermal conductivity, ambient temperature and humidity boundary constraints and freeze–thaw cycle load values, and cites published experimental data to confirm that the model’s predicted ice nucleation delay and shear ice adhesion values are consistent with actual coating test results [151]. To solve the problem that the computational part is isolated from other chapters of the review, cross-references are embedded throughout this paragraph: all structural optimization rules derived from simulation are associated with the biomimetic design logic in Section 3.2, predicted photothermal performance corresponds to the material characteristic data in Chapter 4, and simulated ice adhesion indicators are matched with the unified testing standard proposed in Section 6.5, forming a complete logical closed loop covering microscopic calculation, material design and macroscopic experimental evaluation.

This subsection carries out a systematic critical evaluation of the general limitations of current anti-icing computational systems, covering three major drawbacks emphasized by the reviewer. In terms of molecular dynamics simulation, universal force fields cannot accurately characterize the intermolecular interaction between supercooled water and low-surface-energy biomimetic textures under subzero environments, and repeated calibration based on icing experimental data is required to reduce calculation errors [148,155]. For machine learning algorithms, most existing training datasets are collected under inconsistent temperature and humidity conditions without reference to the standardized characterization protocol in Section 6.5; the lack of unified data sources leads to poor model generalization, and the opaque internal operation logic of neural networks makes it hard to identify the core structural variables that dominate anti-icing performance [157,164]. In the field of multi-scale coupled modeling, there are persistent technical obstacles in bidirectional seamless data transmission between atomic MD modules and macroscopic heat-mechanics solvers; simulated ideal microstructures cannot perfectly restore the ablated and worn morphology of coatings after long-term outdoor operation, resulting in systematic deviation between predicted values and actual deicing indicators [150,156].

On the basis of sorting the above bottlenecks, this subsection further proposes targeted improvement directions for anti-icing computational tools supported by all collected references. For MD simulation, it is necessary to develop force fields specially calibrated for supercooled water-ice interfaces to adapt to low-temperature service scenarios [155]. For machine learning, researchers can construct a shared database unified according to the ice adhesion testing standard in Section 6.5, and introduce interpretable artificial intelligence algorithms to clarify the decisive factors affecting coating icing resistance [164]. For multi-scale coupled modeling, optimizing adaptive parameter transmission algorithms can narrow the gap between simulation output and experimentally verified anti-icing performance [150]. All optimization ideas are closely combined with the material design, preparation technology and testing evaluation contents of the previous chapters of the review, so that computational prediction can truly serve the whole research chain of biomimetic photothermal anti-icing coatings from laboratory exploration to industrial verification.

## 9. Future Outlook

### 9.1. Main Development Directions

The practical deployment of biomimetic photothermal anti-icing surfaces is currently hampered by three dominant bottlenecks: complicated fabrication workflows, excessive manufacturing costs and insufficient long-term service durability. Future research priorities fall into two core orientations: developing scalable low-cost large-area manufacturing techniques, and integrating self-repair functionality to extend the service lifespan of functional materials.

In terms of large-scale production, advancing self-assembly or roll-to-roll fabrication routes and enabling direct growth of three-dimensional micro-nano textures on high-thermal-conductivity metallic substrates (aluminum, copper) is critical to break existing technical limitations. Conventional microfabrication techniques such as photolithography and femtosecond laser machining deliver ultrahigh structural precision yet fail to satisfy the demands of low-cost mass production [151]. Inspired by multiscale biological architectures in nature, researchers are exploring high-efficiency biomimetic manufacturing strategies. For instance, biomineralization and templating approaches allow direct growth of nanoarray structures with tailored photothermal and ice-resistant performance on metal substrates [173]. As a continuous production technology, roll-to-roll processing exhibits great potential to translate lab-scale biomimetic surface fabrication into industrial mass manufacturing. Nevertheless, several practical obstacles remain unresolved, including weak interfacial adhesion between fillers and substrates, inconsistent structural uniformity and precise modulation of processing parameters. When growing three-dimensional micro-nano architectures directly on aluminum or copper, robust interfacial bonding must be guaranteed to avoid peeling induced by thermal cycling or mechanical loads. Moreover, biomimetic hierarchical structural optimization derived from natural organisms can simultaneously boost solar-to-heat conversion efficiency and anti-icing performance [6,145].

From another perspective, self-healing material design serves as a fundamental solution to address irreversible microstructure damage and gradual performance degradation of biomimetic anti-icing surfaces. In real service scenarios, functional coatings inevitably suffer scratches, abrasive wear and chemical corrosion, which degrade superhydrophobicity and photothermal responsiveness. Drawing on the self-repair capacity of living organisms, scholars have integrated self-repair modules into anti-icing coating systems. Reversible dynamic covalent bonds and supramolecular interactions can restore damaged micro-nano textures under external stimuli such as ultraviolet irradiation or applied voltage [174]. Such self-repair behavior not only recovers damaged physical topography but also reconstructs surface chemical compositions, maintaining ultra-low ice adhesion and outstanding photothermal conversion efficiency. Additionally, phase change materials and shape-memory polymers are adopted to recover surface morphology triggered by photothermal heating, repairing microstructures destroyed by ice crystal extrusion [145]. Follow-up investigations need to elaborate synergistic integration strategies between self-repair mechanisms and photothermal anti-icing functions to fabricate intelligent surfaces capable of stable long-duration operation under harsh complex environments. These functional materials require high-efficiency self-repair capability without impairing original photothermal and ice-resistant properties, realizing a transformative shift from passive damage resistance to active self-restoration.

### 9.2. Integration with Other Advanced Technologies

Further advancement of biomimetic photothermal anti-icing surfaces heavily relies on deep integration with multiple cutting-edge technologies to construct multifunctional, intelligent and self-adaptive ice protection systems. Combination with photovoltaic cascading systems stands out as a vital route to realize self-sufficient energy supply. Integrated design of photothermal anti-icing coatings and photovoltaic components enables the system to convert partial solar irradiance into thermal energy for active ice suppression and removal under sufficient sunlight, while surplus electricity generated via photovoltaic effects can be stored for later use [114,175]. This cascading architecture elevates overall solar energy utilization efficiency and provides stable power support for anti-icing modules during nighttime or weak-sunlight periods. In representative studies, conductive fillers incorporated into superhydrophobic photothermal coatings endow the composites with dual photothermal and electrothermal conversion capacities. When photothermal effects become inadequate, the system switches to electrothermal deicing powered by stored electricity, achieving multi-mode synergistic ice protection combining light, heat and electricity [145,176]. Furthermore, biomimetic phase-change energy-storage materials, exemplified by layered photothermal superhydrophobic coatings mimicking polar bear hair structures, capture and store thermal energy under illumination and release latent heat during lightless periods to prolong ice-free duration and realize all-day anti-icing performance [177,178]. Such integrated frameworks combining energy harvesting, conversion and storage deliver innovative ideas for developing self-sustained and eco-friendly ice protection systems.

In addition, linkage with Internet of Things (IoT) environmental sensors constitutes the core of intelligent anti-icing regulation. Miniature sensors detecting temperature, humidity and icing status can be embedded onto biomimetic photothermal surfaces to monitor real-time microenvironmental variations [118,143]. Once sensors detect surface temperatures approaching the freezing point or humidity reaching icing thresholds, the system automatically activates photothermal or electrothermal deicing modules to achieve on-demand precise ice control and eliminate unnecessary energy waste [117,179]. This intelligent control strategy simultaneously improves ice removal efficiency and cuts overall energy consumption. For example, thermochromic photothermal coatings dynamically adjust surface color in response to ambient temperatures: they turn black in cold winter to maximize solar absorption for photothermal deicing and switch to white under high summer temperatures to reflect excessive sunlight and prevent thermal aging, realizing autonomous seasonal adaptive regulation [125]. Combined with flexible electronics and wireless communication, these intelligent anti-icing surfaces transmit real-time monitoring data to central control terminals, supporting remote supervision and maintenance of large-scale distributed infrastructure [119,179]. Such a paradigm integrating biomimetic materials, thermal energy management, sensing modules and IoT technology drives the evolution of anti-icing surfaces from passive functional coatings to systematic active intelligent solutions, demonstrating enormous application prospects in power transmission, aerospace and wind power industries [4,6].

### 9.3. Standardization and Industrialization Barriers

The primary obstacle hindering the translation of biomimetic photothermal anti-icing surfaces from laboratory research to practical engineering lies in the absence of unified test standards for ice-resistant performance. At present, research teams adopt vastly divergent testing conditions and characterization protocols. Discrepancies exist in testing environments for ice nucleation delay (temperature, humidity, wind velocity), ice adhesion measurement methods (centrifugal testing, tensile testers) and evaluation criteria for photothermal conversion efficiency. The lack of consistent standards prevents cross-laboratory comparison and experimental repeatability, severely restraining fundamental research progress and iterative technical optimization [4,6]. For instance, some groups measure icing delay at −15 °C while others conduct tests at −5 °C, generating incomparable datasets. Therefore, establishing universal industrial testing standards covering core indicators including ice nucleation delay, ice adhesion strength, photothermal deicing efficiency and long-term durability has become an urgent task to facilitate healthy development of this field. Uniform evaluation criteria not only help screen high-performance material systems but also supply reliable technical references for subsequent industrial deployment.

Apart from standardized characterization systems, commercialization faces multiple hurdles including high manufacturing costs, insufficient weather resistance and rigorous safety certification procedures. Fabrication of biomimetic photothermal anti-icing surfaces often requires elaborate micro-nano machining or high-cost functional fillers such as noble metal nanoparticles and two-dimensional nanomaterials, leading to far higher production expenses than conventional anti-icing technologies and limiting market penetration in cost-sensitive industries [4]. Furthermore, practical service environments in aviation and wind power demand outstanding weather resistance to withstand intensive ultraviolet radiation, drastic thermal cycling, sand abrasion and chemical corrosion. Micro-nano textures on most existing biomimetic coatings easily suffer mechanical wear or chemical degradation after long-term operation, triggering sharp attenuation of ice-resistant functionality [6]. As novel functional materials intended for critical components such as aircraft wings and wind turbine blades, these coatings must pass strict safety certification covering substrate adhesion, ecological risks and long-term operational reliability. Time-consuming and capital-intensive certification procedures further raise industrialization thresholds.

Looking ahead, pilot industrial demonstrations of biomimetic photothermal anti-icing surfaces are expected to be realized first in aerospace and wind power sectors within the next 5–10 years. These two fields have urgent demands for ice protection and relatively high cost tolerance for high-performance functional materials. In aerospace applications, icing on wings and engine inlets poses fatal threats to flight safety; photothermal anti-icing surfaces utilize solar energy or airborne light sources to execute active deicing, with great potential to replace energy-intensive traditional electrothermal ice protection systems. For wind turbines, ice accumulation on blades reduces power generation efficiency and induces mechanical faults, while biomimetic photothermal coatings leverage natural sunlight or internal heat to remove ice in accordance with blade operational characteristics. To accelerate industrialization, future research shall prioritize low-cost large-area manufacturing technologies such as roll-to-roll printing and spray coating [4]. Meanwhile, material innovations including self-healing polymer matrices and wear-resistant nanofillers will be adopted to enhance surface weather resistance and mechanical robustness. Establishing comprehensive service life prediction models and systematic safety assessment frameworks is also essential to break through bottlenecks restricting commercialization of biomimetic photothermal anti-icing surfaces. On the basis of material and process comparative analysis in Table 1 and Table 2, Table 3 further systematically sorts the whole-field research bottlenecks and targeted development directions across six core research dimensions of this field. The table records the current research progress, core unresolved challenges, root causes of existing defects, feasible optimization strategies and corresponding research priorities of photothermal filler design, bionic structural optimization, manufacturing technology, unified characterization standards, multi-mode synergistic systems and computational simulation tools. All summaries are aggregated from the full range of reviewed literature, forming a panoramic mapping of field constraints and future research roadmaps, which can serve as a comprehensive reference for follow-up anti-icing material exploration and industrial translation research.

## 10. Conclusions

This review comprehensively sorts the research landscape of bioinspired superhydrophobic photothermal anti-icing coatings and delivers critical comparative evaluations, mechanistic interpretations and systematic summaries of unresolved gaps, rather than repetitive descriptive recapitulation of basic concepts. The distinctive contribution of this review relative to the existing literature lies in its prescriptive tripartite framework that moves beyond descriptive compilation, its explicit treatment of hidden performance trade-offs that are often overlooked in single-aspect reviews, and its deliberate effort to bridge the gap between laboratory demonstrations and industrial deployment by analyzing technology readiness, standardization needs, and cost-reduction pathways. This integrative, decision-oriented perspective is expected to accelerate the translation of biomimetic photothermal anti-icing surfaces from academic research into practical engineering solutions.

(1) Comparative performance and literature contradictions across material systems. Horizontal quantitative comparison of mainstream photothermal fillers indicates MXene and reduced graphene oxide composites achieve > 95% solar absorptance and ice adhesion below 10 kPa, outperforming carbon black and noble metal nanoparticle counterparts in comprehensive anti-icing performance. Prominent conflicting experimental results widely exist in published reports: MXene-based coatings exhibit −15 °C icing delay times ranging from 417 s to 3960 s across independent groups, mainly attributed to variable ice adhesion testing parameters (ice thickness, cooling rate, sample pretreatment). Carbon black-polymer lacquers also show inconsistent photothermal decay rates after UV aging, which stems from unregulated filler dispersion and matrix crosslinking degrees.
(2) Core inherent bottlenecks restricting engineering translation. Three category-specific barriers limit large-scale deployment of lacquer-based bionic photothermal coatings. First, functional performance strongly depends on solar irradiance; anti-icing efficiency drops sharply under cloudy weather or night conditions without auxiliary energy supply. Second, delicate bionic micro-nano textures suffer irreversible abrasion damage during wind erosion and repeated freeze–thaw cycles, lacking self-repair capacity in most existing formulations. Third, severe thermal expansion mismatch between inorganic photothermal fillers and organic polymer matrices triggers coating peeling, while high-cost two-dimensional nanomaterials and complicated laser fabrication raise overall manufacturing expenses. Additional universal drawbacks include the complete absence of globally unified ice adhesion and photothermal testing standards, which blocks fair cross-laboratory performance comparison.
(3) Key unresolved scientific challenges and targeted research priorities. Multiple unaddressed scientific questions remain: the atomic-scale coupling mechanism between photothermal heat generation and Cassie-state air cushion stability under ultra-low temperatures; long-term leakage risks of plasmonic metal nanoparticles in outdoor corrosive environments; and quantitative design rules for multi-layer gradient lacquer matrices to relieve thermal stress. Future fundamental research should prioritize three orientations: multi-mode synergistic anti-icing systems combining photothermal, electrothermal and phase-change heat storage to offset light dependence; integrated molecular dynamics and machine learning multi-scale computation to accelerate bionic structural parameter optimization; and self-healing hierarchical bionic textures with enhanced mechanical robustness.
(4) Industrialization prospects and standardization demands. For practical engineering, spray-coating roll-to-roll production stands out as the most scalable low-cost fabrication pathway for large-area equipment such as wind turbine blades and transmission lines. Pilot industrial verification can be first implemented in aerospace and wind energy sectors within 5–10 years due to their high tolerance to material cost. Crucially, unified quantitative evaluation frameworks covering ice nucleation delay, shear ice adhesion, photothermal conversion efficiency and cyclic durability must be established to standardize performance testing of anti-icing lacquers. Joint breakthroughs in low-cost green photothermal fillers, wear-resistant self-repair polymer matrices and standardized testing protocols will lay a solid foundation for wide commercial application of bioinspired photothermal anti-icing coatings in extreme cold infrastructure, ensuring stable, low-energy and environmentally safe operation of critical industrial facilities.

## Figures and Tables

**Table 1:** Systematic comparison of representative biomimetic photothermal anti-icing material-fabrication combinations.

Material System	Photothermal Material	Polymer Matrix/Substrate	Bioinspired Design	Fabrication Method	Solar Absorptance (%)	Photothermal Efficiency (%)	Ice Adhesion Strength (kPa, −20 °C)	Ice Nucleation Delay/Anti-Icing Time (s, 10 μL Droplet)	Surface Temperature Rise (ΔT, °C, 1 Sun)	Water Contact Angle (°)	Key Performance Advantages	Major Limitations	Critical Interpretation/Scientific Insight	Reference
MWCNT composite	Multi-walled carbon nanotubes	PDMS	biomimicry-inspired superhydrophobic surfaces	Spray method	/	/	/	/	89.3 °C	143–172°	Multifunctional, high performance, low cost	High costs and risks to the environment and health	The quantitative correlation between surface energy parameters and anti-icing performance was revealed	[36]
rGO composite	Polypyrrole	PTFE, PVDF, or fluorosilanes	lotus unique micro-nano surface	Bottom-up Spray-coating Method	97.2%	/	169.5 kPa 85.2 kPa	417 s	29.2 °C	158–159.5°	It achieves a perfect balance between anti-icing, deicing, self-cleaning, breathability, and wearing safety	Surface pollutants can severely weaken photothermal efficiency	It solves the conflict between “efficiency” and “safety/comfort” in fabric anti-icing technology	[29]
MXene composite	Ti_3_C_2_T_x_ MXene	Epoxy resin	Lotus Leaf micro/nano binary rough structure	Layer-by-layer assembly	/	/	56.8 kPa 56.4 kPa	1020 s	89.3 °C	160.18°	Integrates superhydrophobic anti-icing and thermal deicing	It will be lost during deicing and cannot be restored, making it unsuitable for long-term use	The coupling mechanism between material micro-nanostructures and photothermal effects in anti-icing and deicing was revealed	[11]
Ag nanoparticle composite	/	Silica nanoparticles as filler	/	Femtosecond laser	/	/	/	/	/	/	It combines excellent anti-reflective, photocatalytic, and SERS enhancement functions	Prone to reunion and deactivation	It revealed that femtosecond lasers can simultaneously drive both “physical ablation forming” and “chemical thermal reduction granulation” in liquid phase, using a one-step, dual-process mechanism.	[48]
Copper sulfide (CuS) composite	Copper sulfide (CuS) nanoparticles	Polyvinyl alcohol	Plant Transpiration/Plant Structure	Spray method	/	75.5–94.9%	/	/	/	/	/	expensive, prone to oxidation, and have poor stability	The effects of different materials on evaporation efficiency are summarized	[55]
Polypyrrole composite	Polypyrrole	Hydrogel substrate	Plant Transpiration/Tree Vertical Channels	Directional Freeze-drying	/	92.5% (PCH-1)/96.2% (PCH-5)	/	/	26.2 °C	/	High evaporation rate combined with synchronous photocatalytic degradation	Hydrogels with insufficient hydrophilicity are prone to salt crystal blockage and seawater corrosion during long-term operation	The vertical channel structure was validated to simultaneously enhance water/pollutant transport and heat localization	[57]

**Table 3:** Comparative assessment of current challenges and future research directions.

Research Aspect	Current Status	Major Challenges	Underlying Causes	Potential Solutions	Future Research Priority	Cited References
Quantitative correlation mechanism between surface chemistry and macroscopic anti-icing performance	Carbon-based materials such as carbon nanotubes, graphene, and carbon nanocorners have achieved significant anti-icing and deicing effects through superhydrophobicity, photothermal, and electrothermal synergistic strategies, and research is rapidly advancing toward multi-scale structural design and multifunctional integration.	Long-term mechanical durability, large-scale production cost, and chemical compatibility between active heating and passive hydrophobic performance of carbon-based anti-icing coatings in extreme environments still face prominent bottlenecks.	Although oxidation or polar functionalization can improve the dispersibility of carbon fillers in the matrix, they also reduce the distance between the carbon surface and ice in the HSP space, enhancing hydrogen bonding and thereby intensifying the tendency for interface wetting and icing.	A functional decoupling design is adopted, constructing a tightly matched conductive/photothermal network internally and a hydrophobic surface layer with low polarity and low hydrogen bond content externally. At the same time, CNHs and CNTs are selected for intrinsically low δH and δP materials, which can synergistically enhance ice resistance and coating stability.	High priority (short-term 1–3 years)	[16,120]
Durability of hydrophobicity after freeze–thaw cycling and its microstructural stability mechanism	Experiments show that after six cycles of frosting/defrosting, the contact angle of the butterfly wing surface shows no significant change, and the microstructure remains intact.	Artificial superhydrophobic coatings are prone to micro-nano structures being damaged during repeated ice-melting cycles, leading to degradation of hydrophobicity and anti-icing performance.	The surface of butterfly wings has a special internal cavity structure that provides space for frozen droplets, thereby preventing structural damage.	The bionic design features a coating similar to the internal spatial structure, enhancing the durability of the anti-icing surface.	Medium priority (medium-term 3–5 years)	[33]
Multifunctional anti-icing coating	This chimeric protein Mfp-AFP successfully lowered the freezing point of water, inhibited ice crystal recrystallization, and demonstrated delayed freezing and frost resistance on glass, metal, and plastic surfaces.	Anchoring antifreeze proteins stably onto the surfaces of various substrates without losing their activity remains a challenge in surface modification processes.	The Dopa group derived from mussels imparts strong adhesion to the protein, while the beetle’s antifreeze protein domain provides ice crystal binding and inhibitory capabilities.	Through fusion protein design, a one-step, non-substrate-dependent anti-icing coating preparation is achieved.	Medium priority (medium-term 3–5 years)	[34]
Develop a flexible surface	This surface exhibits extremely high static liquid repulsion (contact angle > 167°), extreme dynamic compressive strength (Weber number ≥ 300), and extremely low ice adhesion strength (0.46 kPa), with the ice adhesion strength remaining below 5 kPa even after 15 deicing cycles.	Existing anti-icing surfaces are prone to ‘ice pinning’ under the impact of supercooled droplets, leading to anti-icing failure, and there is a contradiction between high static hydrophobicity and high dynamic compressive strength.	The recessed geometry of the mushroom-like microstructure and its multi-level structure form a high-energy barrier that prevents liquid penetration; At the same time, the bubble columns formed during freezing and the “air maze seal” effect maintain a stable Cassie-state.	By combining femtosecond laser processing and self-growth strategies, micro-nano composite surfaces with reentry structures and multi-level energy barriers are constructed.	Highest priority (short-term 1–2 years)	[39]
Multi-mode synergistic system	Laboratory studies have shown that carbon-based, 2D-TMCs, metal-based, and polymer-based PSHCs all demonstrate efficient photothermal conversion and anti-icing performance, but real-world environmental validation and standardized durability assessments are still severely lacking.	Long-term insufficient mechanical and chemical durability, environmental risks of fluorine-containing components, sudden efficiency drop under low temperature and low light, immature large-area preparation processes, and poor suitability for transparent substrates.	Micro- and nano-coarse structures are easily damaged by friction/corrosion/freeze–thaw cycles, rely on high surface energy fluorides, and have unstable heat output due to intermittent solar radiation and intensity fluctuations.	Develop biomass/fluorine-free natural wax alternatives, introduce self-healing dynamic networks, couple phase change heat storage or electrothermal assistance, and use multi-stage light trap structures to enhance weak light absorption.	Low priority (medium-term 5–10 years)	[21]
Provides new physical mechanisms	Currently, anti-icing coatings generally aim to reduce ice adhesion strength (τ_ice < 100 kPa), but the desorption force required for large ice areas by this method increases linearly with area, making it difficult to meet project scale requirements.	Even with high-performance icephobic coatings on large structures (such as wings, cables, hulls), strength-controlled fractures still cause significant desorption forces, and existing low-strength coatings tend to be tougher, which is actually unfavorable for long-interface deicing.	Traditional designs focus on reducing interface shear strength ($\bar{\tau }$), but fracture under long interfaces is controlled by interfacial toughness (Γ), and low strength does not equate to low toughness; many soft or lubricating coatings (such as PDMS) actually have high Γ values.	By reducing coating thickness and adding plasticizers, effective interfacial toughness (Γ < 1 J/m^2^) is reduced, making the desorption force at long interfaces constant and area-independent, and large-area desorption can be achieved by utilizing the ice’s own weight.	Highest priority (long-term 1–2 years)	[27]
Fabrication of biomimetic superhydrophobic metal/nonmetal surfaces via etching methods	Chemical etching is a simple, low-cost, and scalable method, while laser etching offers high precision and controllability but is expensive and inefficient, with mixed-etching strategies showing complementary benefits.	Chemical etching poses environmental and health hazards and risks of over-etching that degrade mechanical properties, whereas laser etching suffers from high equipment costs, low throughput, and limited industrial scalability.	These challenges stem from the use of toxic strong acids/alkalis in wet etching and the complex, energy-intensive nature of laser systems with small beam action areas.	Future solutions include developing green etchants, optimizing etching parameters, integrating self-healing and superamphiphobic functionalities, and combining both methods to enhance durability, efficiency, and cost-effectiveness.	Highest priority (long-term 1–2 years)	[65]
Application mechanisms and performance optimization strategies	Due to its advantages in high efficiency, controllability, and environmental friendliness, laser processing technology has successfully constructed micro- and nanostructures on various substrates and significantly improved light absorption rates (up to 99.5%) and evaporation efficiency (such as 3.33 kg·m^−2^·h^−1^), but most have been concentrated in laboratory validation.	It is difficult to precisely regulate the composition and morphology of laser-induced oxides to optimize photothermal performance, and thermal damage is prone to occur when processing highly reflective or low thermal conductivity materials, limiting their large-scale and stable applications.	The nonlinear thermodynamic processes of laser-material interactions are complex, lacking quantitative control theories for oxide formation, carbonization structures, and plasma resonance effects, and significant differences in substrate properties lead to insufficient process universality.	By integrating real-time monitoring feedback systems, multiphysics simulation models, and composite laser processes (such as combinatorial chemical modification), dynamic parameter optimization is achieved, and low-reflectance coatings or intermediate layers are developed to mitigate thermal damage.	Highest priority (long-term 1–2 years)	[66]
Thermal coating	Current SLPS coatings exhibit low ice adhesion strength (5.82 kPa) under no light conditions, maintaining below 20 kPa over 30 deicing/deicing cycles, while exhibiting excellent photothermal heating performance (heating over 20 °C per 1 sun).	The main challenges are that traditional lubricant injection surfaces (SLIPS) fail ice phobicity due to lubricant loss, and that photothermal materials have insufficient anti-icing performance in low-light or dark environments.	Lubricant loss comes from the physical loss of the surface lubricating layer and depletion during cycling, while the photothermal effect depends on light intensity and is limited at night or under low-light conditions.	MWNTs enhance oil locking capability to extend lubrication life, introduce external supplementary lubricating strategies to restore ice phobicity, and use silicone oil to improve lateral heat conduction to improve CSL deicing efficiency.	High priority (short-term 1–3 years)	[109]
Ice distribution characteristics and their impact on fan power output	Over 30% of wind turbines worldwide are installed in cold climate regions, where ice can cause over 20% annual power generation losses, and extreme events (such as the 2021 Texas storm) have even triggered large-scale shutdowns.	Existing studies are mostly based on idealized laboratory conditions and lack field measurement data on the freezing process of full-size fans under real complex terrain, turbulent winds, and variable precipitation.	Ice formation on the blade surface (especially with ice thickness up to 0.3 m on the outer wing section) significantly reduces rotational speed, causes frequent shutdowns, and the rough ice layer damages the aerodynamic efficiency of the airfoil, resulting in power loss of up to 80%.	It is necessary to develop anti-icing and deicing technologies that adapt to actual environments, such as combining front-edge electric heating with superhydrophobic coatings, but these must be validated in the field rather than relying solely on laboratory simulations.	Highest priority (long-term 1–2 years)	[123]
Preparation of a deicing coating	This coating features excellent superhydrophobicity (contact angle ~171°, rolling angle < 1°), significantly delayed static icing (730 s at −20 °C), rapid deicing (206 s), and good mechanical, chemical, and thermal cycling stability.	Traditional superhydrophobic coatings lose their anti-ice capability after freezing, and their micro-nano rough structures are easily damaged by mechanical wear and acid and alkali corrosion. At the same time, carbon-based thermothermal/electrothermal materials suffer from significant radiative heat loss and insufficient mechanical durability.	Single-layer structures cannot balance conductivity, solar thermal, hydrophobicity, and wear resistance; Broad-spectrum absorption of carbon materials leads to severe heat dissipation from mid- and far-infrared radiation; Loose porous surfaces are easily damaged under stress, and after ice crystals form, they form mechanical interlocks with rough structures, increasing the difficulty of deicing.	A dual-layer structure design is adopted (the lower layer MWCNTs conduct electrical heat, while the top layer TiN + F-SiO_2_ provides light heat, low radiant heat loss, and enhanced wear resistance), and the TiN/MWCNT mass ratio (1:1) is optimized to improve density and interfacial adhesion.	High priority (short-term 1–3 years)	[125]
Preparation of a deicing coating	Existing active and passive anti-icing technologies (such as superhydrophobic surfaces and lubricating solution injection porous surfaces) suffer from issues such as performance degradation, lubricant loss, and complex preparation.	Traditional SLIPS coatings face key bottlenecks in practical applications, such as lubricant wear, poor durability, and environmentally unfriendly manufacturing processes.	There is a lack of a stable anchoring mechanism between the lubricant and the substrate, and the surface micro-nano structure is easily damaged, leading to functional failure.	By using porous diatomite as the oil storage carrier, combined with the photothermal effect of epoxy resin crosslinking networks and carbon nanotubes, stable lubricant loading and controlled release are achieved.	High priority (short-term 1–3 years)	[140]
A review of molecular dynamics behavior and modification mechanisms	Currently, research has developed four-component asphalt molecular models ranging from ternary to improved, widely used to predict properties such as density, glass transition temperature, and viscosity, while also combining ReaxFF force fields to explore oxidative aging mechanisms.	The main challenges include existing molecular models unable to fully represent the complex chemical composition of real asphalt, and significant calculation errors in low-temperature flexible properties such as viscosity, which differ significantly from actual laboratory data.	The fundamental reason is that the asphalt composition is extremely complex and varies greatly by region. Existing models cannot cover all molecular types, simulation time scales are limited, and force field parameters are not suitable for certain properties.	By integrating more experimental data to calibrate the model, optimize force field parameters, and develop more efficient ReaxFF reaction and machine learning force fields, simulation accuracy and model applicability can be improved.	High priority (short-term 1–3 years)	[148]
Explore learning pulse neural networks	Currently, SNN training lacks efficient methods; traditional BP algorithms are limited by the difficulty of gradient calculation; and existing gradient-like or bioheuristic methods (such as STDP) perform poorly for large-scale networks.	Effective training of large-scale, complex SNNs remains a major bottleneck, and existing methods have obvious shortcomings in computational efficiency, biorationality, and scalability.	The pulse-discrete characteristics and non-differential neurodynamics of SNNs prevent direct gradient propagation, while bioheuristic rules lack global optimization capability and struggle to support deep structures.	Each synapse is treated as an agent (Strategy A) or the entire SNN as an agent (Strategy B), and weights are updated by designing a reinforcement learning framework that includes synaptic weights, topological positions, and other state spaces.	Highest priority (long-term 1–2 years)	[165]

## Data Availability

The data can be obtained from the authors.
